# Experimental coral reef communities transform yet persist under mitigated future ocean warming and acidification

**DOI:** 10.1073/pnas.2407112121

**Published:** 2024-10-29

**Authors:** Christopher P. Jury, Keisha D. Bahr, Annick Cros, Kerri L. Dobson, Evan B. Freel, Andrew T. Graham, Rowan H. McLachlan, Craig E. Nelson, James T. Price, Mariana Rocha de Souza, Leah Shizuru, Celia M. Smith, Wesley J. Sparagon, Cheryl A. Squair, Molly A. Timmers, Jan Vicente, Maryann K. Webb, Nicole H. Yamase, Andréa G. Grottoli, Robert J. Toonen

**Affiliations:** ^a^Hawai’i Institute of Marine Biology, School of Ocean and Earth Science and Technology, University of Hawai’i at Mānoa, Honolulu, HI 96744; ^b^Department of Life Sciences, Texas A&M University—Corpus Christi, Corpus Christi, TX 78412; ^c^The Nature Conservancy, Arlington, VA 22203; ^d^Marine Biology and Ecology Research Group, School of Ocean and Earth Sciences, University of Southampton, Southampton SO14 3ZH, UK; ^e^School of Earth Sciences, College of Arts and Sciences, The Ohio State University, Columbus, OH 43210; ^f^Department of Microbiology, Oregon State University, Corvallis, OR 97331; ^g^Daniel K. Inouye Center for Microbial Oceanography, Research and Education, Department of Oceanography and Sea Grant College Program, School of Ocean and Earth Science and Technology, University of Hawai’i at Mānoa, Honolulu, HI 96822; ^h^Department of Biology, School of Life Sciences, College of Natural Sciences, University of Hawai’i at Mānoa, Honolulu, HI 96822; ^i^Department of Marine Biology, College of Natural Sciences and School of Ocean and Earth Science and Technology, University of Hawai’i at Mānoa, Honolulu, HI 96822; ^j^Pristine Seas, National Geographic Society, Washington, DC 20036

**Keywords:** coral reef, climate change, ocean acidification, mitigation

## Abstract

Coral reefs are among the most sensitive ecosystems affected by ocean warming and acidification, and are predicted to collapse over the next few decades. Reefs are predicted to shift from net accreting calcifier-dominated systems with exceptionally high biodiversity to net eroding algal-dominated systems with dramatically reduced biodiversity. Here, we present a two-year experimental study examining the responses of entire mesocosm coral reef communities to warming (+2 °C), acidification (−0.2 pH units), and combined future ocean (+2 °C, −0.2 pH) treatments. Contrary to modeled projections, we show that under future ocean conditions, these communities shift structure and composition yet persist as novel calcifying ecosystems with high biodiversity. Our results suggest that if climate change is limited to Paris Climate Agreement targets, coral reefs could persist in an altered state rather than collapse.

## Significance

Coral reefs are exceptional ecosystems and support hundreds of millions of people around the world, yet they are under severe threat due to ocean warming and acidification. Reefs are predicted to collapse over the next few decades under these climate change stressors, with grave consequences for society. Contrary to predictions of near total destruction, this study shows that with effective climate change mitigation, coral reefs will continue to change, but global reef collapse may still be avoidable.

Many studies predict that the combination of ocean warming and acidification will lead to the functional collapse of coral reef ecosystems at a global scale over the next few decades, driving major losses in biodiversity and ecosystem services (1–6). Corals are expected to be essentially extirpated from reefs in the next few decades (>99% decline) (6–8) if ocean temperatures warm by an additional 0.5 to 1 °C (about 1.5 to 2 °C above the preindustrial) and coral bleaching becomes a nearly annual phenomenon. Likewise, reefs are expected to shift from net calcification to net carbonate dissolution sometime later this century if atmospheric CO_2_ reaches 550 to 650 µatm, seawater pH declines by 0.1 to 0.15 units below present-day (about 0.2 to 0.25 below the preindustrial), and seawater temperature increases by 1 to 1.5 °C above present-day (about 2 to 2.5 °C above the preindustrial) (4, 9). These projected future scenarios are typically based on either short-term laboratory perturbation experiments with few species that are then scaled up to long-term responses of complex communities in nature (3, 4, 10, 11) or in situ observations of coral reefs that span natural gradients in thermal stress or chemistry (12–15). Laboratory experiments, however, typically do not encompass the natural diversity in species responses or ecological interactions among species which could affect community outcomes, and many experiments may not have provided organisms with sufficient time to fully respond to treatment conditions. Further, adaptation may improve the responses of many of these organisms over coming generations. Likewise, natural gradient studies do not reflect the intensity of heat stress expected later this century, they often do not incorporate warming and acidification simultaneously [but see (16–18)], and they almost never account for variation in water flow or other environmental parameters which are known to affect coral responses to heat stress (19). Nonetheless, short-term lab experiments and natural gradient studies, as well as the modeling results based on them, provide our current best understating of how reefs may change in the future. An alternative approach to lab or natural gradient studies is the use of mesocosms which allow biologically diverse communities to be exposed to future ocean levels of warming and acidification while preserving ecological interactions among species as well as realistic environmental conditions (20–25). While no study can incorporate every aspect of future reef ecosystem composition and species interactions, high-quality mesocosms provide one of the most complete experimental systems to test the responses of coral reef communities to sustained future ocean levels of warming and acidification.

We conducted a two-year outdoor flow-through mesocosm experiment to examine the long-term responses of biologically diverse coral reef communities to chronic warming and acidification similar to a century worth of global change at current warming rates (26). It is important to note that these levels of warming and acidification will co-occur under the transient climate response, given a high CO_2_ emissions scenario (SSP5-8.5) in about the year 2075 (27). However, the level of acidification will diminish over time as the Earth system responds to this pulse of CO_2_ and moves toward equilibrium, such that acidification levels will only be about half of the levels imposed here around the year 2400 and will diminish to about one-third of these levels in the year 3000 (28, 29). In contrast, temperatures will remain at nearly the same levels for the next millennium (29). These levels of warming (about 3 °C above the preindustrial) and acidification (about 0.3 pH units below the preindustrial) exceed the international targets set under the Paris Climate Agreement of no more than 2 °C of warming and −0.2 pH units of acidification relative to the preindustrial. Despite widespread concern, efforts to achieve these international targets remain slow and will require international cooperation and substantial reductions in current CO_2_ emissions (26). Even with global agreement to commit to CO_2_ emission reductions, temperatures will continue to rise such that reaching or even exceeding these targets now seems inevitable.

To examine the impacts of expected near-future climate change on coral reef communities, mesocosms were initially stocked with replicate communities of the regionally most common reef-building corals, as well as a homogeneous mix of reef rubble, reef sand, algae, invertebrates, and fish (see Supplementary Information). Corals were each sourced from multiple locations around the island of O’ahu, Hawai’i. We determined the multilocus genotype of each coral colony sampled to ensure results were not biased by inclusion of clones. From these genetically distinct coral colonies, replicate clonal fragments (ramets) of each genetically unique coral colony (genet) were included in all four treatments and fed with unfiltered natural seawater from the adjacent coral reef ecosystem. Over time, the mesocosms recruited a diverse assemblage of algae, invertebrates, and microbes. These communities developed under one of four treatments with 10 mesocosms per treatment: control treatment (present-day temperature and pH), ocean warming treatment (elevated temperature of +2 °C relative to control and present-day pH), ocean acidification treatment (present-day temperature with acidification of −0.2 pH units relative to control), or combined future ocean treatment (both elevated temperature of +2 °C and acidification of −0.2 pH units relative to control) (*SI Appendix*, Fig. S1). All mesocosms experienced natural diurnal and seasonal variation in temperature, chemistry, and irradiance (Table 1 and *SI Appendix*, Fig. S2). After approximately two years of exposure, we assessed i) coral survivorship, 3-D cover, recruitment, and physiology ii) net calcification by the mesocosm communities, coral communities, and rubble-associated communities, and iii) community structure and species richness of the major functional groups, including benthic algae and invertebrates, coral-associated algal endosymbionts (Symbiodiniaceae), coral-associated microbes, and water column-associated microbes (*SI Appendix*, Fig. S3). This study included eight coral species representing both major evolutionary lineages of scleractinians (Complexa and Robusta), three globally important reef-building coral families (Acroporidae, Pocilloporidae, and Poritidae), all four of their major life history strategies, as well as many of the dominant coral types across the Indo-Pacific (18, 30). Further, the responses of dozens of species of algae, hundreds of animals, and thousands of microbes were surveyed in our study. Combining all these approaches, we present a two-year experimental examination of coral reef organismal and community responses under future ocean conditions. This dataset provides insights into the potential responses of coral reef communities to ocean warming and acidification over the coming years.

**Table 1.** Seawater carbonate chemistry and temperature from the experiment

Treatment	Salinity (psu)	Temperature (°C)	pH	Total alkalinity (µmol kg^−1^)	pCO_2_ (µatm)	Ω_arag_
Control	34.26 ± 0.34 (0.02)	25.08 ± 1.18 (0.09)	7.99 ± 0.05 (0.01)	2,177 ± 51 (12)	448 ± 60 (12)	2.88 ± 0.32 (0.05)
Ocean acidification	34.26 ± 0.34 (0.02)	25.09 ± 1.18 (0.09)	7.78 ± 0.07 (0.02)	2,184 ± 49 (12)	794 ± 132 (44)	1.94 ± 0.31 (0.08)
Ocean warming	34.29 ± 0.34 (0.02)	26.99 ± 1.18 (0.17)	7.98 ± 0.05 (0.01)	2,187±49 (12)	453 ± 61 (16)	3.06 ± 0.33 (0.07)
Future ocean	34.30 ± 0.34 (0.02)	27.03 ± 1.15 (0.16)	7.77 ± 0.07 (0.02)	2,196 ± 48 (9)	811 ± 134 (45)	2.07 ± 0.32 (0.08)

Data are daily mean values derived from weekly sampling at 1200 h as well as monthly sampling every 4 h over the diel cycle (*SI Appendix*, Supplementary Information) and are shown as mean ± SD. The uncertainties associated with these values reflect daily and seasonal variability, as well as variability among replicate mesocosms in each treatment. The mean uncertainties among mesocosms on a given sampling day are provided in parentheses. Note that the variation among mesocosms is relatively small and most of the variation is explained by daily and seasonal fluctuation of these parameters. *SI Appendix*, Fig. S2 for additional environmental information.

## Results

### Environmental Conditions.

We exposed these communities to target environmental conditions (including +2 °C of warming and −0.2 pH units of acidification) (Table 1 and *SI Appendix*, Fig. S2). This resulted in severe bleaching stress of about 24 DHW (Degree Heating Weeks) accumulated annually in each of 2016 and 2017 by the coral and broader reef communities in the heated treatments. This level of heating was beyond the 15 to 22 DHW accumulated on the Great Barrier Reef, in the Florida Keys, and in many other locations during the unprecedented 2023–2024 marine heat wave (31).

### Coral Genotyping.

Each of the coral colonies sampled exhibited a unique multilocus genotype. Even when allowing for possible scoring errors with the microsatellite markers, none of the corals showed evidence of having been clonally derived. Hence, each coral colony constituted a separate genet consisting of 22 genets for *Montipora flabellata* and 30 genets for each of the remaining seven species (*Montipora capitata*, *Montipora patula*, *Porites compressa*, *Porites evermanni, Porites lobata*, *Pocillopora acuta*, and *Pocillopora meandrina*) yielding a total of 232 coral genets, each of which was examined across all four treatments.

### Coral Survivorship and 3-D Cover.

**Fig. 1.**
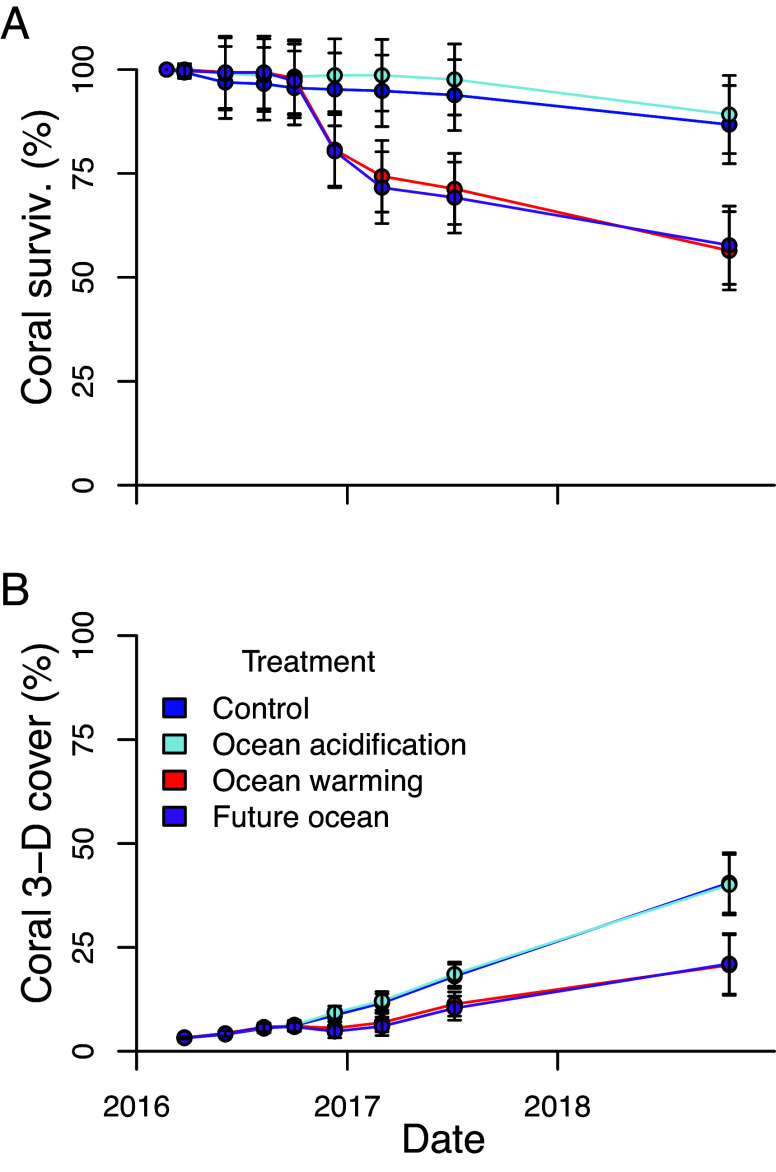
Coral community survivorship and 3-D coral cover. Plots show time series of coral survivorship (*A*) and live 3-D coral cover (*B*) over the course of the experiment according to treatment. Coral cover (the area of skeleton covered by live tissue) is shown as a percentage of the interior surface area of the uncolonized mesocosms (bottom, walls, and standpipe, 0.88 m^2^). Where individual lines and points are not visible, it is because they are overlapping. Data shown as mean ± SD (n = 10 mesocosm communities per treatment). Both coral survivorship and the rate of increase in coral 3-D cover were reduced under warming and neither responded to acidification. *SI Appendix*, Table S2 for test results.

Coral survivorship was reduced by about 35% in the heated treatments (ocean warming and combined future ocean) relative to that in the nonheated treatments (control and ocean acidification) (Fig. 1 and *SI Appendix*, Table S1), though effects were highly variable among species (*SI Appendix*, Fig. S4). Coral 3-D cover increased over time in all treatments, but the rate of increase in the heated treatments (ocean warming and combined future ocean) was only about half of that in the nonheated treatments (control and ocean acidification) (Fig. 1 and *SI Appendix*, Fig. S5). Initial 3-D coral cover was about 3% in all treatments and at the end of the experiment was about 40% in the control and ocean acidification treatments, and about 21% in the ocean warming and combined future ocean treatments (Fig. 1 and *SI Appendix*, Fig. S5). In contrast, acidification had no significant effect on either coral survivorship or 3-D cover in these experiments (Fig. 1 and *SI Appendix*, Figs. S4 and S5 andTables S1 and S2).

### Community Calcification.

**Fig. 2.**
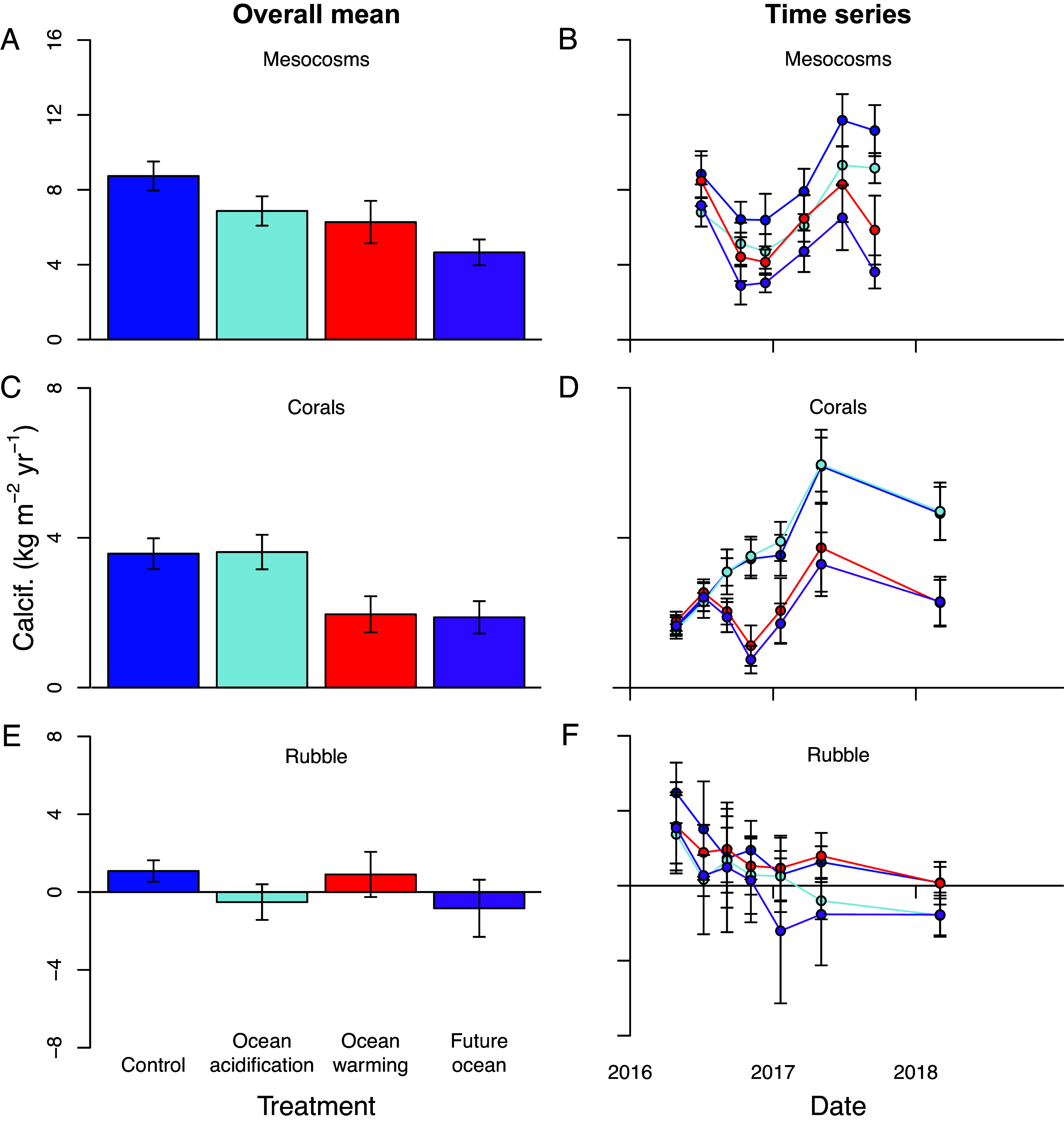
Effects of experimental warming, acidification, and combined future ocean conditions on net community calcification. (*A* and *B*) Entire mesocosm communities (sometimes referred to as net community calcification, NCC, or net ecosystem calcification, NEC, in other studies), (*C* and *D*) coral communities, and (*E* and *F*) rubble-associated communities. Net community calcification was determined by the total alkalinity anomaly technique in (*A* and *B*) and by the buoyant weight technique in (*C*, *D*, *E*, and *F*). Time-integrated overall mean values are shown on the left (*A*, *C*, and *E*) whereas time series are on the right (*B*, *D*, and *F*). Data bars and points show the mean ± SD (n = 10 mesocosm communities per treatment). Mesocosm calcification declined with acidification and warming, whereas coral calcification declined only with warming and rubble calcification declined only with acidification. *SI Appendix*, Table S2 for test results.

Entire reef mesocosm communities exhibited a significant reduction in net calcification rate due to both warming and acidification, though these factors did not interact (Fig. 2 and *SI Appendix*, Table S2). In contrast, coral communities showed reduced net calcification under warming, yet did not respond to acidification, while rubble-associated communities displayed reduced net calcification under acidification and did not respond to warming (Fig. 2 and *SI Appendix*, Table S2).

### Community Structure and Species Richness.

**Fig. 3.**
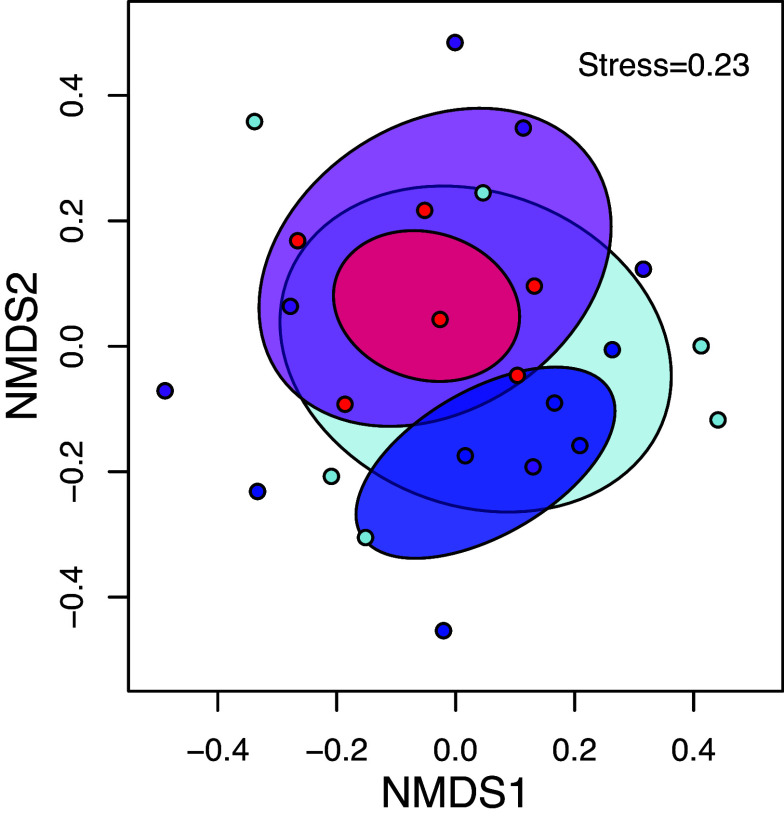
Benthic community structure in the mesocosms at the end of the study. Nonmetric multidimensional scaling ordination illustrating the effects of ocean warming and acidification on benthic community structure from settlement tiles (ARMS) colonized in the mesocosms for the control (blue), ocean acidification (light blue), ocean warming (red), and combined future ocean treatments (purple) (n = 6 tile arrays per treatment). Scatterplots are overlaid on ellipses which show the SD around the centroid for each group. Ellipses are semitransparent to allow visualization of overlapping distributions. Community structure differed according to temperature but not pH, which was driven largely by separation of the ocean warming treatment from the control. Fig. 4 and *SI Appendix*, Table S3 for additional results.

**Fig. 4.**
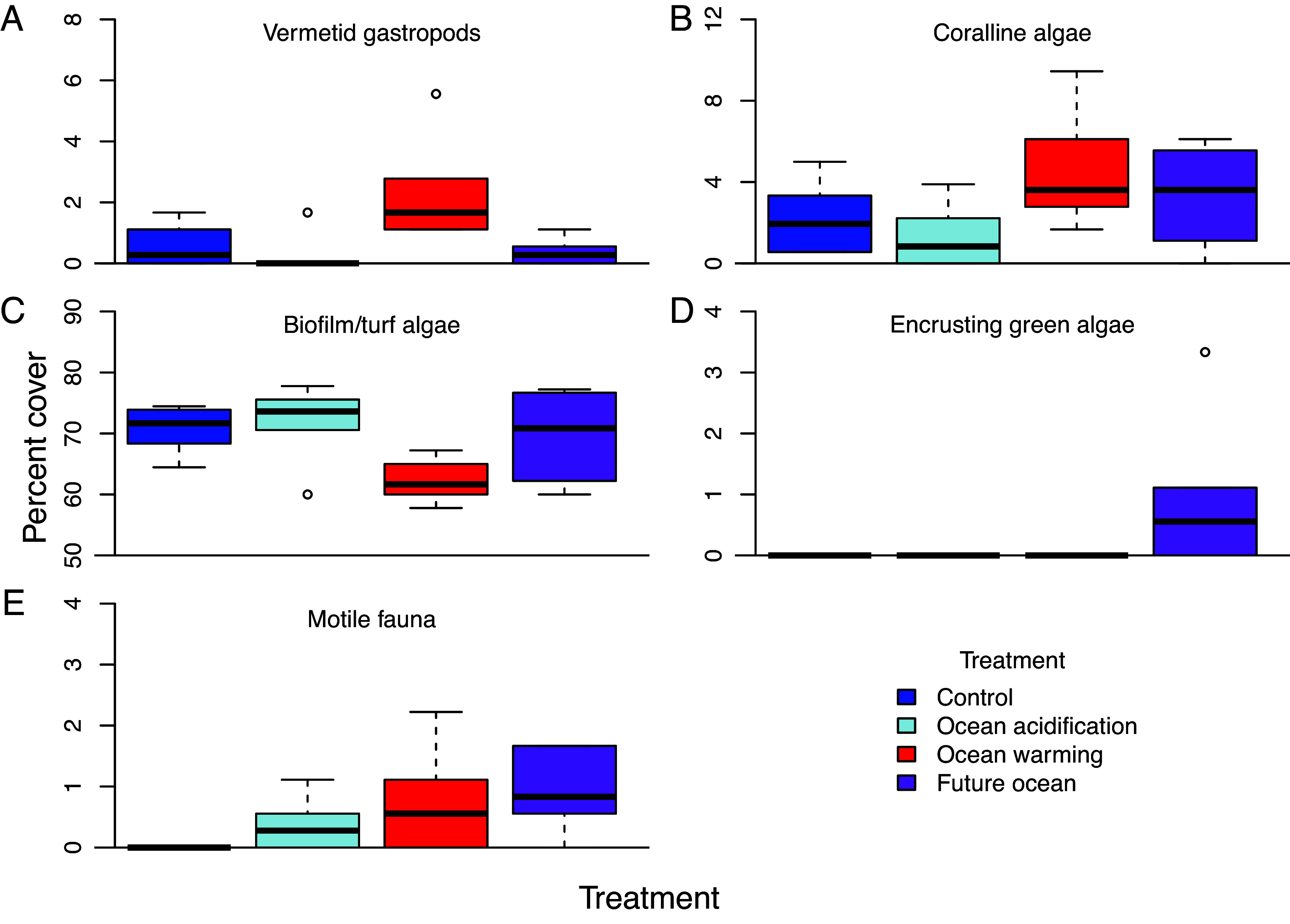
Benthic cover of functional groups on settlement tiles colonized in the mesocosms at the end of the study. (*A*), vermetid gastropods (*B*), crustose coralline algae (CCA), (*C*), biofilm/turf algae, (*D*), encrusting green algae, (*E*), motile fauna (n = 6 ARMS tile arrays per treatment). Vermetid gastropods, CCA, biofilm/turf algae, and motile fauna showed significant responses to warming, whereas only vermetid gastropods responded negatively to acidification. Encrusting green algae attained significantly higher abundance under the combined future ocean scenario as compared to the other treatments. None of the other functional groups (anemones, bivalves, macroalgae, sediment, serpulid worms, sponges, tunicates, and uncolonized space) responded significantly to treatment conditions. Box-plots show the median as center line, box limits are upper and lower quartiles, whiskers are 1.5× interquartile range, and open circles as outliers. Boxes are not visible where values were below detection limits of 0.09%. *SI Appendix*, Tables S2 and S4 for test results.

Noncoral benthic community structure on settlement tiles [modified Autonomous Reef Monitoring Structures, ARMS (32)] differed significantly according to temperature, but not by pH (Fig. 3 and *SI Appendix*, Table S3). While community structure tended to be more variable in the acidified treatments (ocean acidification and combined future ocean), dispersion was not significantly different among treatments. This separation in community structure was explained by responses among a subset of the functional groups on the settlement tiles (Fig. 4 and *SI Appendix*, Tables S2 and S4). We considered the power of these analyses, and at a power of 0.95, the minimum effect size which we can detect is about 1.69, or a difference in benthic cover among treatments of about 0.5%. Vermetid gastropod cover increased under warming yet declined under acidification. Crustose coralline algae (CCA) cover increased under warming, whereas biofilm/turf algae declined under warming, but neither responded to acidification. Encrusting green algae cover increased under the combined future ocean treatment. Finally, the abundance of motile fauna (primarily amphipods and brittle stars) increased under warming. None of the other functional groups (anemones, bivalves, fleshy algae, sediment, serpulid worms, sponges, tunicates, and uncolonized space) differed in percent cover occupied among the treatments (*SI Appendix*, Tables S2, S4, and S5).

**Fig. 5.**
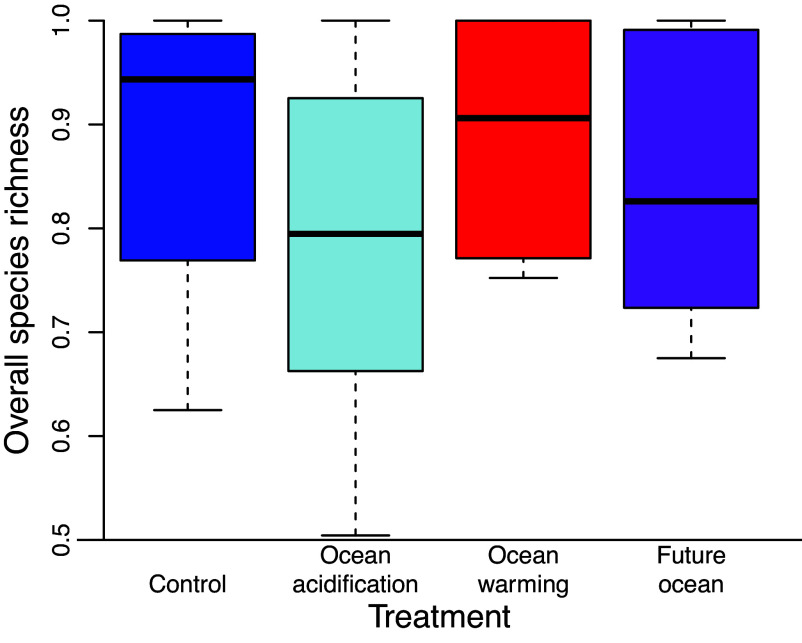
Overall species richness in the mesocosms at the end of the study. Treatment effects on overall species richness in the mesocosms (data shown as proportional variation in species richness relative to the maximum observed richness among treatments) derived from samples of sponges, crustose coralline algae (CCA), and metabarcoding of metazoans from settlement tiles (ARMS), coral-associated microbes, water column-associated microbes, coral-associated algal endosymbionts, fleshy algae, and corals (n = eight datasets representing dozens of species of algae, hundreds of types of animals, and thousands of types of microbes). Box-plots show the median as center line, box limits are upper and lower quartiles, whiskers are 1.5x interquartile range, and there were no outliers. Treatment effects were not significant.

Coral species richness was reduced under heating, primarily due to the loss of one or both *Pocillopora* spp., but was unaffected by acidification (*SI Appendix*, Table S2). In contrast, coral-associated microbe (33) richness increased under heating but was unaffected by acidification, whereas sponge species richness exhibited the opposite pattern and declined under reduced pH but did not respond to warming (*SI Appendix*, Table S2). Cryptic metazoans (crustaceans, echinoderms, snails, worms, etc.) inhabiting settlement tiles (ARMS), on the other hand, exhibited increased richness under warming and reduced richness under acidification (32). None of water-column-associated microbes, coral-associated algal symbionts, crustose coralline algae (CCA), or fleshy algae richness differed significantly among the treatments (*SI Appendix*, Table S2). Finally, when these eight datasets were combined (and weighted equally by dataset) to examine changes in the overall species richness of these communities, none of the treatments differed significantly (Fig. 5 and *SI Appendix*, Table S1).

## Discussion

We created biologically diverse mesocosms with predicted ocean warming (+2 °C) and acidification (−0.2 pH units) supplied with unfiltered seawater from the adjacent reef to examine the coral reef communities that developed over the course of two years. The IPCC Climate Change 2023 Report concludes with very high confidence that coral reefs will decline by >99% under these future ocean conditions (27), but we observed comparatively less severe responses than have been projected. Rather than collapsing into extremely low coral cover, net carbonate dissolution, and markedly reduced biodiversity under future ocean conditions, these communities instead transitioned into novel calcifying reef systems with diminished yet substantial coral cover and maintained high biodiversity. Reef communities that developed in each of warming, acidification, and the future ocean combination of both factors, showed substantial changes in community structure and composition relative to the community that developed under present-day conditions. Reefs of the future will certainly be different from those of today (34, 35)

### Coral-specific Responses.

Corals in the treatments with elevated temperature (both ocean warming and combined future ocean treatments) were exposed to severe (3) heat stress in successive years. These corals experienced temperatures at or above the nominal bleaching threshold for the Main Hawaiian Islands for 3.5 mo per year, during which they accumulated 24 degree heating weeks (DHW) annually (Figs. 1 and 2 and *SI Appendix*, Figs. S1, S4, and S5). Many studies predict that this level of repeated annual bleaching stress should have been sufficient to nearly extirpate corals in our elevated temperature mesocosms (1, 3, 7, 8) and that acidification should have exacerbated the heat stress (3). The accumulation of 15 to 22 DHW on the Great Barrier Reef, in the Florida reef tract, and in many other locations worldwide during the unprecedented 2023–2024 marine heatwave has resulted in devastating consequences for some corals and other organisms. Likewise, many of the corals that bleached severely in this study subsequently died (Fig. 1 and *SI Appendix*, Fig. S4 andTable S1). However, contrary to projections of near total mortality (7, 8), coral survivorship was reduced by an average of only 35% in the heated treatments compared to the present-day temperature treatments, and with no evidence that acidification affected survivorship (Fig. 1 and *SI Appendix*, Fig. S4 andTable S1). The effects of heating on survivorship, however, differed dramatically among coral species. While *P. meandrina* suffered catastrophic mortality (97 to 100%) in the heated treatments, *P. evermanni* exhibited high survivorship (92 to 95%) regardless of heat stress. The remaining six species exhibited intermediate levels of survivorship with a general tendency toward higher survivorship among *Porites* spp., intermediate survivorship among *Montipora* spp., and lower survivorship among *Pocillopora* spp. Indeed, while some individual corals in this study bleached and died following the first heat stress event, others bleached annually yet survived to the end, and still others never bleached at all.

Despite severe annual heat stress in the heated treatments, live coral cover increased substantially over the course of the experiment in all four treatments, from about 3% at the beginning to about 40% in the control and ocean acidification treatments, and about 21% in the ocean warming and combined future ocean treatments by the end of the study (Fig. 1 and *SI Appendix*, Fig. S1 and Table S2). Hence, the rate of increase was reduced by about half in the heated treatments (ocean warming and combined future ocean) as compared to the nonheated treatments (control and ocean acidification). This decrease resulted from lower coral survivorship, partial mortality, and reduced growth rates among many of the survivors under heating. Similar to the survivorship responses, acidification had no significant effect on live coral cover.

After nearly two years of exposure under treatment conditions, we examined the physiological performance of a subset of the corals. This subset was chosen based on logistical constraints about how many coral ramets could possibly be measured within a reasonable timeframe and we focused on the three most common species (*M. capitata*, *P. compressa*, and *P. lobata*). Among the survivors, *M. capitata* exhibited reduced carbon budgets under heating, indicative of stress, whereas *P. compressa* experienced enhanced photosynthesis under acidification, while *P. lobata* did not differ physiologically among treatments (36). Part of these physiological responses appear to be related to the coral-associated microbiome (33). Surviving *M. capitata* genets had a distinct, seemingly advantageous, microbial community composition compared to genets that died. In contrast, the microbial community composition associated with each of *P. compressa* and *P. lobata* shifted in response to the treatments. These results suggest that some of the corals (but not necessarily others) experienced a degree of physiological and microbial acclimatization to the treatments over the course of the study. Indeed, coral species exhibit substantial variability in their capacity to acclimatize to heat stress (37). After the conclusion of the mesocosm experiment, we tested for the possible effects of temperature preconditioning on future performance within two of the coral species included in this study (*M. capitata* and *M. flabellata*) (38). Replicate coral ramets that were maintained under ocean warming conditions for more than two years were compared to those maintained under present-day conditions throughout, and their performance was assessed before and during a natural coral bleaching event in an open ocean nursery. Over four years of consecutive heat stress events, we found no evidence that preconditioning provided either long-term temperature acclimatization and resistance or sensitization to future bleaching for either species, because responses were indistinguishable based on temperature history during subsequent transplantation to a common garden. Differences in growth rate and survival in each species were driven by individual genotype of the corals, rather than preconditioning to thermal stress (38). These results were confirmed by a recent field survey wherein individually tagged colonies of *M. capitata* exhibited no long-term change in thermal tolerance over multiple, natural bleaching events (39). In contrast, *P. compressa*, which we included in the mesocosm study but were unable to include in the field trial, does exhibit increased thermal tolerance over the course of years under repeated marine heatwaves (39). Thus, acclimatization is unlikely to increase long-term thermal tolerances for all corals and adaptation via natural selection must play an important role in future responses for some (30).

Large numbers of one coral species (*P. acuta*) recruited into the mesocosms due to spawning of the adult corals housed within them (20, 40), but recruitment rate was unaffected by any of the treatments (40). Recruitment only occurred in mesocosms which contained live adults of the species and the lack of treatment effects on recruitment might be related to the weedy life history strategy exhibited by this coral. Indeed, *P. acuta* is effective at rapid colonization and can often persist in marginal habitats, despite the fact that it was one of the most thermally sensitive species examined here. We also observed widespread spawning of one of the competitively dominant coral species (*M. capitata*) across all four treatments (CPJ pers. obs. in June 2018). Hence, at least some of these corals were reproductive under future ocean levels of warming and acidification. Unfortunately, it was infeasible to assess reproductive output or gamete quality at the time of release. Early life stages are often especially vulnerable to environmental stressors, such as heating and acidification. If the larvae and recruits of these coral species exhibit more severe responses to warming and acidification than do the adults, then our results may underestimate the coral decline which should be expected later this century. While our observations of recruitment are limited to one species in this study, if coral individuals that are resistant to these conditions (like some of those in this study) proliferate in the future, then they could help to reduce the decline in coral abundance predicted for coral reefs under future ocean warming and acidification. Indeed, coral communities in Hawai’i already appear to be mounting adaptive responses to climate change with bleaching and mortality occurring at higher temperatures and after longer exposures than reported 50 y ago (18, 41), and all eight of the coral species examined here exhibit clear scope to adapt to ocean warming, ocean acidification, and the combination of both factors (30, 42).

### Community Calcification.

The calcification rates measured in the control mesocosm communities were very similar to those measured on the nearby reefs (43), suggesting that the mesocosms adequately replicated the processes involved in community calcification. Net calcification of the mesocosm communities (sometimes referred to as net community calcification, NCC, or net ecosystem calcification, NEC, in other studies) declined in all treatments relative to the control, with the largest decline under the combined future ocean scenario (Fig. 2 and *SI Appendix*, Table S2). The 19 to 24% reduction in mesocosm calcification attributable to acidification is lower but roughly similar to the 30% reduction measured on an experimentally acidified reef flat (44, 45), further illustrating the efficacy of our approach to simulate the natural system. Nevertheless, all communities continued calcifying. Even under the combined future ocean treatment, reef community calcification was positive, albeit at only 56% the rate of control reef communities. At present-day rates of calcification, however, few reefs are expected to accrete fast enough to be able to keep up with sea level change and many future reefs may become submerged as the oceans rise (46). Indeed, even under the scenario we present here, many reefs are likely to be drowned or move shoreward given the rising ocean (34, 35, 46)

Acidification does not by itself kill corals but rather tends to inhibit their skeletal growth by an average of 15 to 20% (11), which may compromise their competitive abilities in nature (12, 13, 16). Acidification, however, had no effect on net coral community calcification within this study (Fig. 2 and *SI Appendix*, Table S2). While these results differ from many prior laboratory experiments, both ex situ and in situ studies have found that some corals can maintain normal calcification rates under lower pH (13, 14, 18, 47–49). Further, irradiance and water flow are both known to affect coral responses to acidification (21). We conducted this experiment using natural sunlight (attenuated by shade cloth to ambient levels at mean collection depth of 2 m), rapid turnover (1 h) with unfiltered natural seawater, and additional water circulation provided by seawater pumps (10 to 15 cm s^−1^) to replicate light and flow conditions on the natural reefs as closely as possible (*SI Appendix*, Figs. S1 and S2). These more natural conditions may help to explain the observed insensitivity of coral calcification to low pH relative to many previous laboratory studies, which rarely replicate natural reef conditions (50). In addition, corals may show threshold responses to acidification such that they are able to maintain calcification rates under a 0.2 pH unit reduction yet experience reduced calcification rates at higher levels of acidification (18, 20, 30). Unlike acidification, elevated temperature reduced coral community calcification by nearly half due to bleaching, mortality, and reduced growth among the survivors (Fig. 2 and *SI Appendix*, Table S2). In contrast, net calcification by rubble-associated communities declined under ocean acidification conditions yet was insensitive to warming (Fig. 2 and *SI Appendix*, Table S2). These results suggest that the measured reductions in calcification for mesocosm communities (24, 25, 51) and natural communities (9, 44, 52) due to acidification are driven largely by processes occurring within the reef framework rather than by the corals themselves. This may result in the reef being more brittle and more vulnerable to storm damage. The calcification budget of the mesocosms exceeded that explained by the corals and rubble, and this additional carbonate production was likely from the growth of crustose coralline algae (CCA) and other organisms which formed thick, calcified crusts on the mesocosm walls. These crusts were slowly eroded and regrown over time in a continuous cycle over the course of the experiment.

Future reefs will undoubtedly experience a major decline in growth due to the loss of corals from heat stress, and reduced calcification of the reef framework under acidification. Nonetheless, these communities continued calcifying at about half of present-day rates under potential future ocean conditions. The maintenance of live coral cover along with the growth of CCA and other calcifiers within all mesocosms helped to support net calcification by these communities.

### Community Structure and Species Richness.

Corals are ecosystem engineers, yet coral reef biodiversity is derived largely from the array of algae, invertebrates, and microbes which live within, among, and upon the reefs. Coral reefs occupy less than 0.2% of the seafloor but are home to an estimated 32 to 38% of all marine species (53). Yet the vast majority of research is focused on a handful of reef-building coral species (50), and almost nothing is known about how this biodiversity will respond to ocean warming, acidification, or the combination of both factors. To determine how algal, microbial, and noncoral invertebrate composition varied within each treatment, at the end of the experiment we 1) retrieved 3-tiered settlement tile arrays [modified Autonomous Reef Monitoring Structures (54)] which had recruited diverse benthic assemblages while soaking in the mesocosms for the duration of the experiment; 2) sampled the coral-associated algal endosymbionts and coral-associated microbes, as well as the water column-associated microbes; and 3) sampled the mesocosms for both CCA and benthic, fleshy algae. The settlement tile arrays mimicked the three-dimensional structure of the reef framework (albeit not the microstructural complexity of the reef) and provided a standardized tool with which to examine this often-overlooked yet highly diverse cryptobenthic community. Given the relatively short reproductive cycles of many algae and invertebrate species (weeks to months), they experienced multiple generations over the course of the experiment, providing a time-integrated measure of the treatment effects on community composition and abundances. Throughout the course of the experiment the benthic community transitioned from early colonizing species to a mature and diverse community that underwent seasonal variation in abundance similar to adjacent reef communities (55).

Benthic cover analyses of the settlement tiles by functional group revealed that community structure differed only by temperature, and this effect was driven largely by separation of the control and ocean warming treatments in a community ordination (Fig. 3 and *SI Appendix*, Table S3). Indeed, only a subset of the functional groups responded significantly to the treatments. Calcifying vermetid gastropods declined under acidification but increased under warming (Fig. 4 and *SI Appendix*, Table S2). Calcifying CCA cover also increased with warming (Fig. 4 and *SI Appendix*, Table S2). The CCA crusts in the ocean acidification treatment were noticeably thin as compared to the other treatments but the trend toward lower cover was nonsignificant. Metabarcoding revealed that the biomass of CCA and other red algae was dramatically reduced under acidification alone, yet these algae reached maximum biomass under the combined future ocean treatment, suggesting a compensatory interaction between warming and acidification (32). In contrast, the cover of noncalcifying biofilm/turf algae decreased with warming, but was likewise unaffected by acidification (Fig. 4 and *SI Appendix*, Table S2). This response may be an indirect effect of reduced space availability as some other groups increased their abundances under warming. Encrusting green algal cover was below detection limits in most of the treatments but achieved modest abundance in the combined future ocean scenario (Fig. 4 and *SI Appendix*, Table S4). Finally, the abundance of motile fauna (consisting primarily of calcifying amphipods and brittle stars) increased under warming and exhibited a nonsignificant trend toward higher abundance due to acidification (Fig. 4 and *SI Appendix*, Table S2). Results from visual surveys were confirmed through metabarcoding wherein the abundance of most members of these motile groups increased substantially due to both warming and acidification (32). The higher abundance of these organisms under warming and acidification might result from differences in detritus production (55), which serves as a primary food source for these animals. The benthic cover of the other functional groups on the settlement tiles (anemones, bivalves, macroalgae, sediment, serpulid worms, sponges, tunicates, and uncolonized space) did not respond to the treatments (Fig. 4 and *SI Appendix*, Tables S2, S4). However, metabarcoding revealed that all four treatments exhibited distinct community structure (32). These data show that community composition shuffles, with some species losses offset by others’ gains, depending on treatment, and these differences were not detected at the coarser level of functional group.

Taxonomic and functional groups showed highly variable responses to the experimental treatments (Fig. 4), complicating predictions about exactly how reefs will respond to future ocean conditions. However, when all taxonomic datasets (corals, sponges, metabarcoding of metazoans from settlement tiles, CCA, fleshy algae, coral-associated microbes, water column-associated microbes, and coral-associated algal endosymbionts) were pooled to examine the effects of elevated temperature and reduced pH on proportional changes in overall biodiversity, the number of species was not significantly affected by any of the treatments (Fig. 5 and *SI Appendix*, Table S1).

## Conclusions

Perhaps the most important question that emerges from this work is why these results contrast with many earlier projections about reef futures under global change. We believe that there are three especially important factors which help to explain these discrepancies. First, previous studies have focused on a small subset of the natural diversity of species-specific and genotype-specific coral responses to global change with half of published research focused on just three coral species (*Acropora millepora*, *Pocillopora damicornis*, and *Stylophora pistillata*) (50). In line with many projections, the *Pocillopora* species in this study (*P. meandrina* and *P. acuta*) suffered greatest mortality under heating. In contrast, many other coral species, and particularly some individual genotypes within these species, showed less severe responses under warmer and more acidic conditions than has been projected. Second, these communities exhibited reduced calcification due to both warming and acidification, but did not transition to net carbonate dissolution. Projections of future reef decalcification rely on both the chemical effects of acidification (which we can confirm) as well as the predicted near total loss of corals, which did not occur here. Instead, corals and some other calcifiers exhibited greater persistence under future ocean conditions than has often been assumed, reflecting the natural diversity in their responses. In particular, CCA have have been shown to acclimatize to ocean acidification over the course of months (56, 57) and these taxa exhibited their highest performance under the combined future ocean treatment in this experiment. Persistence of these calcifying taxa in the mesocosms allowed these communities to maintain net calcification. Third, many projections are based on single factor studies of warming or acidification showing negative impacts on corals. While combinatorial experiments remain relatively less common, it has often been assumed that most reef organisms will exhibit similarly negative outcomes under warming and acidification as compared to corals. However, very few other reef organisms have ever been investigated for their responses under future ocean conditions. Indeed, many reef organisms are yet to be formally described or named, much less investigated for their responses to global change (32). While coral species richness did decline under ocean warming and was unresponsive to acidification, most other functional groups exhibited fundamentally different responses to each factor as compared to the corals. These discrepancies may reflect our lack of knowledge about most of these species, but also likely reflects a failure to account for the complexity of species interactions within biologically diverse communities.

These reef communities persisted when exposed to chronic warming and acidification for two years, yet they were fundamentally transformed and, in several ways, they were diminished. Our results emphasize the critical importance of mitigating both climate change and the intensity of local stressors. Without effective climate change mitigation, reefs will become increasingly degraded, yet it seems likely that coral reefs will face severe environmental stress, even under the best-case climate change scenario. Without effective local management, corals will be unable to recolonize damaged reefs (18, 58). The treatments we imposed here are on par with a business-as-usual climate change scenario of +2 °C warming above present-day (about +3 °C above the preindustrial) and 0.2 pH units below present-day (about 0.3 below the preindustrial). If the world achieves Paris Climate Agreement targets of limiting climate change to no more than 2 °C above the preindustrial, then most reefs will rarely. if ever, experience the intensity of heat and acidification stress that we imposed here (59). Overall, our results imply that with effective climate change and local stressor mitigation, reef communities will continue to change, but global collapse of coral reefs may still be avoidable.

## Materials and Methods

### Approach.

The experiment was conducted at the University of Hawai’i at Mānoa, Hawai’i Institute of Marine Biology (HIMB) on Moku o Lo’e (Coconut Island), immediately adjacent to the island of O’ahu in the Hawaiian Archipelago, starting on 1 February 2016. Most samples and other measurements were collected after approximately two years of exposure under treatment conditions (*SI Appendix*, Fig. S3). The mesocosms received constant flow-through of unfiltered seawater drawn from the adjacent coral reef (2 to 3 m depth, depending on tide). The 40 mesocosms were initially stocked with a well-mixed 2 cm layer of carbonate reef sand and gravel as well as pieces of reef rubble collected from the adjacent reef, thereby including natural infaunal and surface-attached communities. In addition, a juvenile (3 to 8 cm) Convict surgeonfish (*Acanthurus triostegus*), an herbivore, and a Threadfin butterflyfish (*Chaetodon auriga*), a predator on noncoral invertebrates, 5 herbivorous reef snails (*Trochus* sp.), the 8 regionally most common reef-building coral species, and settlement tiles consisting of modified 3-plate stacks of Autonomous Reef Monitoring Structures (ARMS) were added to the mesocosms,

In multiple, independent upstream header tanks, temperature was adjusted using commercial aquarium heaters on temperature controllers, and sea water was dosed with CO_2_ gas using high-precision needle valves connected to venturi valves on aquarium pumps to deliver a precisely controlled quantity of CO_2_ gas that was completely dissolved into the sea water before flowing into the mesocosms. The heated treatments were set to remain +2 °C above the control treatment, whereas the acidified treatments were maintained at −0.2 pH units below the control treatment, thereby replicating conditions which may occur later this century. All mesocosms experienced natural daily and seasonal fluctuations in light, seawater temperature, and carbonate chemistry with appropriate offsets according to treatment.

Given the variety of results generated, several different statistical approaches were needed to analyze the data. For each dataset, temperature and pH were treated as fixed factors/effects and header tank was included as either a nested factor (ANOVA, PERMANOVA) or a random effect (generalized linear mixed models, GLMM), as appropriate for each type of analysis. For most datasets, mesocosm was the unit of replication, whereas for a few (coral survivorship, coral-associated algal symbionts), there were multiple observations in each mesocosm, and therefore we included mesocosm (Tank) as an additional random effect in these analyses. For Kruskal–Wallis (ANOVA on ranks), analyses were based on treatment.

Additional details regarding each component of the study are provided in *SI Appendix*, Supplementary Information.

## Supplementary Material

Appendix 01 (PDF)

## Data Availability

Spreadsheets data have been deposited in Figshare (https://dx.doi.org/10.6084/m9.figshare.27115516) (60). All study data are included in the article and/or *SI Appendix*.
