# Supplementary material for: Experimental coral reef communities transform yet persist under mitigated future ocean warming and acidification
Source: Proc Natl Acad Sci U S A. 2024 Oct 29;121(45):e2407112121. doi: 10.1073/pnas.2407112121 (PMC11551444; doi:10.1073/pnas.2407112121)
Supplement: Supplementary file 1 — Appendix 01 (PDF) [file pnas.2407112121.sapp.pdf]

Supporting Information for

Experimental coral reef communities transform yet persist under mitigated future ocean warming and acidification

Christopher P. Jury, Keisha D. Bahr, Annick Cros, Kerri L. Dobson, Evan B. Freel, Andrew T. Graham, Rowan H. McLachlan, Craig E. Nelson, James T. Price, Mariana Rocha de Souza, Leah Shizuru, Celia M. Smith, Wesley J. Sparagon, Cheryl A. Squair, Molly A. Timmers, Jan Vicente, Maryann K. Webb, Nicole H. Yamase, Andréa G. Grottoli, Robert J. Toonen

Christopher P. Jury
Email: jurycp@hawaii.edu

This PDF file includes:

Supporting text
Figures S1 to S5
Tables S1 to S5
SI References

Supporting Information Text

Materials and Methods

Seawater temperature and chemistry

Relative to the offshore source water, seawater temperature and chemistry are naturally modified by reef-associated physical and biogeochemical processes as the water flows through Kāneʻohe bay (1–3). To restore water temperature and chemistry of the incoming seawater close to that of the original source water, all incoming seawater was directed into a 700 L mixing tank where temperature was adjusted using a commercial heat pump on a temperature controller and chemistry was adjusted with small additions of 1.0 N NaOH via a peristaltic pump (+0.1 pH units, +80 µmol kg^{-1} of total alkalinity) to achieve average present-day offshore temperature and chemistry conditions in Hawaiʻi (temperature 23.5-27.5 °C, pH 7.97-8.07, annually). These small adjustments resulted in modifications to the temperature, pH, and total alkalinity in the seawater input which, when combined with the same physical and biogeochemical processes in the mesocosms, allowed us to achieve conditions very similar to those observed on the reef (1, 4, 5). This incoming water was then split off into a series of 130 L header tanks where it was heated or acidified according to treatment, with 2 replicate header tanks per treatment. Temperature of the incoming seawater was adjusted to average seasonal values twice per month in order to follow the seasonal cycle, whereas pH was adjusted as an offset relative to ambient. Given this seasonal cycle, as well as diel variation due to solar heating and irradiance, the mesocosms provided a realistic analog to conditions on the reef.

Two approaches were used to characterize the water temperature and chemistry in the mesocosms. First, water samples were taken from each mesocosm at 1200 hr local time once per week for total alkalinity and spectrophotometric pH, whereas salinity and temperature were measured with a YSI multimeter. All these procedures followed standard protocols (6). The precision of these measurements were: pH ±0.002 units, salinity ±0.01 psu, temperature ±0.01 °C, total alkalinity ±7 µmol kg^{-1}. The accuracies of these measurements are estimated as: pH ±0.005 units, salinity ±0.3 psu, temperature ±0.1 °C, total alkalinity ±7 µmol kg^{-1}. The accuracy and precision of total alkalinity titrations were assessed using Certified Reference Materials (CRMs) obtained from Andrew Dickson (Scripps Institution of Oceanography). Second, the temperature and chemistry measurements as described above, were assessed every 4 hr over the diel cycle once per quarter. For the remaining two months per quarter, bottle samples were taken for pH and total alkalinity only at 1200 and 0000 hr and a pH meter was used to assess pH at the other diel sampling points (1600, 2000, 0400, and 0800 hr). The pH meter was empirically calibrated to the 1200 and 0000 hr pH bottle samples at the time of collection, yielding an uncertainty of ±0.02 units for these sample points. The monthly diel samples were used to assess the hourly temperature and chemistry variation in the mesocosms as well as the daily mean values on those sampling dates. The weekly water samples, along with the empirically derived relationships between the offset of the mesocosm sea water to the incoming sea water measured at 1200 hr and the daily mean values (characterized during the diel sampling), were used to estimate the daily mean parameters for these remaining dates. These estimates yielded the following uncertainties in the calculated daily means: salinity ±0.12 psu, pH ±0.03 units, temperature ±0.17 °C, and total alkalinity ±16 µmol kg^{-1}. The remaining carbonate chemistry parameters were calculated with CO2SYS (7). This sampling protocol yielded data resolution similar to or greater than that used in previous studies (8–10), but over a longer timeframe and at a much higher level of replication.

To reach initial target treatment temperature and pH values at the beginning of the experiment, adjustments were made in 0.5 °C and 0.03-0.05 pH unit increments every 10 days (starting on 1 February 2016 from baseline values that were intermediate between the temperature and pH treatment levels) until target values were reached (20 February 2016). The slow ramp to target temperature and pH during the winter minimized the chances of shocking the communities and was slower than many natural warming events. The design initially included 40 mesocosms (0.5×0.5×0.3 m, 70 L). Prior to this study, growth rates had been measured in only a few of the coral species and some of the corals showed higher skeletal extension rates than we expected (ranging from about 1-10 cm yr^{-1}). We became concerned that the corals would begin to compete aggressively with each other, resulting in unnecessary mortality before the end of the

study. While coral-coral competition is natural, excessive mortality would have compromised some aspects of our study. Starting on 18 November 2017, after 21 months of exposure and much of the data had already been collected, an additional eight (2 per treatment) larger mesocosms (0.5×1.1×0.3 m, 180 L) were added to maintain three coral species (*Montipora flabellata*, *Pocillopora meandrina*, and *Porites evermanni*) selected for removal to prevent direct competition for space among corals in the mesocosms. We selected one species per genus that included one slow (*Porites evermanni*), moderate (*Pocillopora meandrina*), and fast (*Montipora flabellata*) growing species, to minimally impact the initial mesocosm design. None of the corals or the reef communities showed any obvious adverse reactions to this move, as illustrated in the coral-specific time series. Further, because all 40 mesocosms received the same modification, our results are unbiased by treatment.

The mesocosms were randomly assigned to treatment. Seawater inflow rate was adjusted to 1.2 L min^{-1} (70 L mesocosms) or 3.5 L min^{-1} (180 L mesocosms) for a residence time of 1 hr in all mesocosms. Additional water circulation was generated within each mesocosm by submerged seawater pumps with one Maxi-Jet Pro propeller pump (4900 L hr^{-1}) in each 70 L mesocosm and two pumps in each 180 L mesocosm. Flow speeds in the vicinity of the benthic communities (10-15 cm s^{-1}) were estimated from visual inspection of particle tracks and verified with an independent product review of the pumps (11).

<u>Corals, rubble, and fish</u>

The corals and rubble were placed on a plastic grate 6 cm above the bottom soft sediments, to simulate their attachment to hard substrate in nature. The juvenile Convict surgeonfish (*Acanthurus triostegus*) is a generalist grazer on benthic algae whereas the juvenile Threadfin butterflyfish (*Chaetodon auriga*) is a generalist grazer on non-coral invertebrates. Together, the fish provided the essential ecological functions of herbivory and predation in the mesocosms, and at fish biomass values similar to those reported for Hawaiian reefs (12). As the fish grew larger (>10 cm) they were released to the Marine Life Conservation District surrounding Moku o Loʻe, slowly reducing their densities over the course of the experiment. As fish densities were reduced they were rotated randomly from tanks that contained them to tanks without fish every other week (during the night, to minimize stress on the fish), in order to ensure that all mesocosms received similar levels of grazing throughout the experiment. The fish and the mesocosms were supplementally fed 3 g wet weight of frozen mysis or brine shrimp daily, thereby provisioning the fish and wider reef communities with allochthonous (i.e., non-local, imported) zooplankton at a rate similar to that measured in nature(13). Assessing responses by the fish was not a major focus of this study, though they showed no obvious differences in behavior or condition among treatments, consistent with recent work (14).

The eight coral species included in the mesocosms (*Montipora capitata, Montipora flabellata, Montipora patula, Pocillopora acuta, Pocillopora meandrina, Porites compressa, Porites evermanni,* and *Porites lobata*) are the dominant species across the Hawaiian archipelago and collectively comprise >95% of the coral cover on Hawaiian reefs (15, 16). Historically, four of these species were once thought to be Hawaiian endemics (*Montipora flabellata*, *Montipora patula*, *Porites compressa*, and *Porites evermanni*), though more recent analyses have shown that all eight species (or their genetically indistinguishable kin) are in fact widespread across the Indo-Pacific (17–19). These eight species represent both major lineages of reef-building corals (Complexa and Robusta) (20), and all four of the major life history strategies exhibited by corals(21) including competitive (*Montipora capitata*, *Pocillopora meandrina*, and *Porites compressa*), generalist (*Montipora flabellata* and *Montipora patula*, inferred from the ecologically similar *Montipora monasteriata*), stress-tolerant (*Porites lobata* and *Porites evermanni*, with the strategy for *Porites evermanni* inferred from the ecologically similar *Porites lutea*), and weedy strategies (*Pocillopora acuta*, inferred from its ecologically similar sister species *Pocillopora damicornis*). In prior thermal stress events these species have shown differing resistance to bleaching ranging from low (*Montipora flabellata*, *Pocillopora acuta*, and *Pocillopora meandrina*), to moderate (*Montipora capitata*, *Montipora patula*, *Porites compressa*, and *Porites lobata*), to high (*Porites evermanni)* (50). They also include three of the major reef-building coral families worldwide (Acroporidae, Pocilloporidae, and Poritidae) yielding broad ecological and spatial relevance to this study. Corals were collected at 2±1 m depth from a total of six locations around

Oʻahu (4) between 17 August 2015 and 13 November 2015, to help ensure that a representative sample of their phenotypic and genotypic diversity was included in the study. Sampling of each species was restricted to the subset of sites where it was relatively common (3-5 sites per species), and conspecifics were separated by at least 5 m to minimize the chances of accidentally sampling clones or biasing the sampling towards particular microenvironments (4). All 232 coral parent colonies sampled for this study (22 parent colonies for *Montipora flabellata*; 30 parent colonies for each of the other seven species) were genotyped to ensure that they were distinct genets and results were not biased by inclusion of clonally derived colonies. After collection, the corals were allowed 12 weeks to recover and acclimate to the mesocosm system under the same temperature and pH conditions, thereby standardizing their short-term histories prior to beginning the experiment on 1 February 2016. Most genets contributed one ramet (i.e., coral fragment, 3-5 cm) per treatment whereas four genets per species were represented by three replicate ramets per treatment, resulting in 1184 coral ramets total in the experiment. Each mesocosm initially contained 29-30 ramets (3-4 ramets per species, each of which was a distinct genet) and an initial 3-D coral cover of about 3%.

Irradiance and bleaching threshold

Irradiance data were obtained from the HIMB weather station (https://www.pacioos.hawaii.edu/metadata/AWS-HIMB.html?format=fgdc) at mean hourly resolution. Sunlight was attenuated with 30% shade cloth to provide irradiance similar to that at mean collection depth (2 m) with a maximum instantaneous irradiance of about 1730 μmol m^{-2} s^{-1} in the mesocosms and 2470 μmol m^{-2} s^{-1} in the air. A handheld quantum meter was used to take periodic spot checks to ensure that the desired level of shading was achieved. Light spectrum was not adjusted due to the trivial differences between 0.3 m (mesocosm depth) and 2 m (mean collection depth). The nominal coral bleaching threshold (27.98 °C) was estimated as the mean monthly maximum temperature for the Main Hawaiian Islands (26.98 °C) (22) +1 °C.

Coral genotyping

Multilocus genotyping of coral hosts was performed following published methods (23, 24). Briefly, total genomic DNA was isolated using the E.Z.N.A. Tissue DNA Kit (Omega Bio-tek, Inc., Norcross, GA, USA) following the manufacturer protocol. Amplicons were generated via PCR using microsatellite primers (25), but with short unique barcodes(26) added to each primer to identify each position in a 96 well plate. Amplicons were pooled equimolarly, and a dual-index system of adaptors was used to identify individuals on each plate and libraries were sequenced on an Illumina MiSeq platform (v3 2x300 PE) at the Hawaiʻi Institute of Marine Biology. We used a custom bioinformatic genotyping workflow pipeline (23) to call alleles, which were then converted to GenoDive v. 2.0b27 (27) file format for analyses. Individual genotypes were created using two different methods. First, we used sequence length (equivalent to peak calling in a microsatellite fragment analysis *sensu* (28)), such that all sequences of the same length, regardless of underlying sequence variation, would be scored as the same allele (sequence length). Second, we identified alleles by their sequence (ID) so that only two exactly identical alleles had the same ID, whereas alleles with the same length but differing in nucleotide composition would have different allelic IDs. Similar to previous findings (23), both approaches gave the same result. Using the 'assign clones' feature of GenoDive(29), we tested whether coral colonies sampled in the field had a unique multilocus genotype. To be conservative, we allowed for up to 2 scoring errors among individuals and checked potential clones against the location of collection. Only *Porites compressa* had a pair of colonies (colony #1 and #3 from Waimānalo) that could not reject clonality with 2 scoring errors, yet these colonies exhibited distinctive coloration (yellow-grey vs. tan) and morphology (smoother vs. knobbier branches) which they maintained while growing in a common garden with the other colony for 2 years. This gave us confidence that none of the corals were clonally derived and that all 232 parent colonies represent distinct genets.

Coral survivorship, 3-D cover, and species richness

At the beginning of the experiment, all corals appeared healthy and had normal pigmentation. Survivorship was assessed visually about every 2 months during the first year of the experiment, and at lower frequency thereafter. Survivorship was defined as corals which

either did or did not possess any visible live tissue. Partial mortality was assigned according to the following categories where survivorship was: 100, 95, 90, 80, 70, 60, 50, 40, 30, 20, 10, 5, 0%.

Coral 3-D cover was estimated empirically for each coral genotype based on images from four sides (front, back, left, and right sides) using ImageJ(30) and assuming that skeletal elements were approximately rounded in cross section relative to the 2-D images. The surface area estimates were used along with buoyant weight data (as described below) to produce empirical surface area:weight estimates for each coral genotype. Live coral cover was then determined in combination with the partial mortality scores, as described above.

One live coral went missing before the end of the experiment (probably due to dislodgement and burial by crabs that recruited into the mesocosms) so it was dropped from the analysis. The survivorship data were analyzed by fitting a generalized linear mixed model (GLMMs) with binomial error distribution with temperature and pH as crossed, fixed effects and header tank and mesocosm as random effects. Significance of each factor was evaluated via likelihood ratio tests (LRTs). Pairwise contrasts by treatment were fit using the Tukey method. Coral 3-D cover was assessed via ANOVA with temperature and pH as fixed factors, and header tank as a nested factor, normalized to the internal surface area of the uncolonized mesocosms (0.88 m^2). Coral species richness was analyzed via ANOVA with temperature and pH as fixed factors and header tank as a nested factor, followed by a TukeyHSD *post hoc*. Analyses were performed using the R packages lme4 and lsmeans in R v.3.5.2 (31–33).

Coral, rubble, and mesocosm calcification rates

Coral community and rubble-associated community calcification rates were assessed via the buoyant weighing technique(34). Initial weights were taken in spring 2016, about every 2 months during the first year of the experiment, and at lower frequency thereafter. The original 40 mesocosms provided the coral communities with 0.25 m^2 of horizontal space. Moving the three coral species to the larger mesocosms provided each set of corals with an additional 0.11 m^2 of space (0.36 m^2 total). A subset of the corals was sampled for the physiological and microbial analyses after nearly 2 yr of exposure to treatment conditions. For the microbial analyses, small samples were removed from the corals with bone cutters, whereas for the physiology analyses, fragments were cut from each ramet using a diamond-coated band saw. The corals were weighed before and after fragmentation to determine the skeletal mass removed. None of the corals showed any obvious adverse reactions to fragmentation and they tended to overgrow the wound sites within a couple weeks. Net calcification rates for these corals were then estimated assuming that the fragments removed would have calcified at rates proportional to the remainder of the colony retained in the experiment. In addition to the one live coral that went missing (as mentioned above), four which had previously died also went missing before the end of the experiment (again, likely due to dislodgement and burial by crabs), Therefore, all five of these corals were dropped from the coral community calcification analysis.

Rubble calcification rates were averaged for the three replicate pieces per mesocosm and normalized to planar surface area of the rubble. Planar surface areas were estimated from photographs using ImageJ (30). Two pieces of rubble were accidentally damaged during the experiment, so they were dropped from the analysis.

Whole mesocosm calcification rates were assessed via the total alkalinity anomaly technique(35). Briefly, calcification rates were determined from the alkalinity influx into the mesocosms (in the incoming sea water) minus the alkalinity efflux from them (in the outgoing sea water), divided by 2, given the stoichiometry of carbonate precipitation/dissolution. Seawater flow-through rate was determined by measuring the amount of time needed to fill a container of known volume (875 ml) with a stopwatch (about 45 s). Alkalinity, pH, temperature, and salinity were assessed as above every 4 hr for a 24 hr period (i.e., 1200, 1600, 2000, 0000, 0400, 0800 hr) once per quarter (summer, autumn, winter, spring) from summer 2016 to autumn 2017, resulting in 6 sampling points per day and 6 sampling days total. Daily net calcification rates were taken as the mean of the 6 daily measurements, and the average calcification rate for each mesocosm was taken as the mean of the 6 sampling days, normalized to the planar surface area of the mesocosms (0.25 m^2), thereby taking into account seasonal variation in community calcification

rates. These daily mean values were scaled to annual values by multiplying by 365 d yr^{-1}. To assess the precision of these estimates we propagated the error associated with the total alkalinity titrations (7 µmol kg^{-1}) and seawater flow-through rates (1.6 L hr^{-1}). The resulting uncertainties were 0.12 g d^{-1} and 0.19 g yr^{-1}, or 2-4 orders of magnitude smaller than the estimated calcification rates.

All calcification data were analyzed via ANOVA with temperature and pH as fixed factors, header tank as a nested factor, and mesocosm as the unit of replication, followed by a Tukey HSD as a *post hoc*. Assumptions of normality and equality of variance for ANOVA were assessed via diagnostic plots of the residuals. Analyses were performed using R v.3.5.2 (33).

Coral algal endosymbionts

For Symbiodiniaceae identification, small samples were taken from each surviving coral in November 2018 and preserved in dimethyl sulfoxide, ethylenediamine tetraacetic acid, saturated salt (DESS) solution (36)n. Depending on survivorship, up to 12 corals per species per treatment were randomly selected. Coral fragments had their DNA extracted using E.Z.N.A Tissue DNA Kit. Symbiodiniaceae ITS2 amplicon library preparation and sequencing followed the protocol outlined(37). Briefly, the ITS2 region was amplified for each sample, pooled and sequenced on an Illumina MiSeq platform (v3 2 x~ 300 bp PE) at University of Hawai‘i at Mānoa. Raw sequences were demultiplexed and quality filtered using Cutadapt(38). Forward and reverse reads were submitted to SymPortal(39), a platform for genetically identifying Symbiodiniaceae using high throughput ITS2 sequence data that differentiates intra- and intergenomic sources of ITS2 sequence variance.

In this study, we analyzed data based on Symportal outputs for Symbiodiniaceae “profile”. Symbiodiniaceae profile is a set of ITS2 sequences that have been found in a sufficient number of independent samples to be identified as a ‘defining intragenomic variant’ (DIV). Symbiont richness was assessed via ANOVA in R with temperature and pH as fixed factors, and header tank and mesocosm as nested factors.

Settlement tiles (ARMS plates)

Three-tiered stacks of settlement tiles (modified Autonomous Reef Monitoring Structures, ARMS) were deployed in the mesocosms in July 2016 and retrieved in June 2018 to assess structure and species richness of the benthic communities, with six arrays per treatment (three arrays per replicate header tank) which were assigned randomly to a subset of the mesocosms. Upon retrieval the stacks were disassembled, photographed, subsampled for sponges and CCA (for barcoding and morphological identification), and then scraped of all accumulated biomass for metabarcoding.

Benthic cover: The upper and lower face of each plate was photographed and the images were loaded into CoralNet (https://coralnet.ucsd.edu/) for benthic cover analysis. Thirty stratified random points were overlaid on each image and the benthic cover was identified to functional group (e.g., sponge, vermetid gastropod, CCA, etc.), resulting in 180 points per unit. Treatment effects on overall community structure were assessed via PERMANOVA with temperature and pH set as fixed factors, and header tank nested within them in vegan (40). Since only temperature effects were significant, we examine the treatments using the R package pairwise.Adonis (https://github.com/pmartinezarbizu/pairwiseAdonis) as a *post hoc* and a Benjamini-Hochberg correction for multiple tests was applied. Dispersion was examined among treatments via PERMDISM in vegan. To examine treatment effects on functional groups we used two approaches. First, we took a multivariate approach with the R package indicspecies (41). Second, we took a univariate approach and the proportion data were arcsine square root transformed and an ANOVA was fit with temperature and pH as fixed factors and header tank as a nested factor, followed by a TukeyHSD *post hoc*. For those groups where the data violated the assumptions of ANOVA, a Kruskal-Wallis ANOVA on ranks was fit instead, followed by a Dunn’s test *post hoc* with a Benjamini-Hochberg correction. Both approaches provided similar answers in that the benthic cover of encrusting green algae, biofilm/turf algae, and vermetid gastropods were among the most important drivers of the differences in community structure among treatments. Power analyses were conducted for these groups using the R package pwr (42)

Sponges: Settlement tile units were disassembled into individual plates for subsampling of sponges as detailed previously (43, 44) and summarized below. Each plate face was carefully observed for sponges showing unique morphologies in each unit. Additional sponge samples showing limited characters with the naked eye per unit were also collected. Sponge individuals measuring 0.5 cm or more were photographed and subsampled from each unit using a scalpel. Samples were fixed in 95% ethanol. When enough tissue was available, samples were also fixed in 4% paraformaldehyde and 4% glutaraldehyde in 0.1M sodium cacodylate with 0.35M sucrose for subsequent histological evaluation. Duplicate samples of every sponge species were deposited at the Florida Museum of Natural History.

DNA was extracted from samples fixed in ethanol using the Promega E.Z.N.A. Tissue DNA Kit, following the manufacturer's instructions. PCR was used to amplify partial fragments of the 28S rRNA and COI mtDNA gene sequences. COI amplification first used primers COI1490F (GGT CAA CAA ATC ATA AAG ATA TTG G) and COXR1 (GCG ACT ACA TAA TAA GTR TCR TG) (45, 46). For those samples that did not yield a product, we used a second primer set, jgLCO1490F (TIT CIA CIA AYC AYA ARG AYA TTG) and jgHCO2198R (TAI ACY TCI GGR TGI CCR AAR AAC A) (47), then the forward primer mlCOIintF (GGW ACW GGW TGA ACW TWT AYC CYC C) (48) paired with the primer jgHCO2198R mentioned above. A similar format was used for 28S rRNA analysis starting with the primer pair 28F63mod (ACC CGC TGA AYT TAA GCA TAT HAN TMA G) and 28R1072 (GCT ATC CTG AGG GAA ACT TCG G) (49). For those samples that did not yield a product we used a second pair of primers 28S-C2-fwd (FAA AAG AAC TTT GRA RAG AGA GT) and 28S-D2-rev (TCC GTG TTT CAA GAC GGG) (50). All PCR reactions were carried out in 40 µL total volume including the following: 14.49 µL of H2O, 20 µL of BioMixTM Red (Bioline, Taunton, MA, USA) PCR Mastermix, 0.8 µL of each primer (10 mM), 3.2 µL of BSA (100 mg mL^{-1}), and 0.85 µL of template DNA (5-50 ng μL^{-1}). The PCR programs all consisted of an initial denaturation at 94 °C for 3 min followed by 34 cycles of 94 °C for 30 s. Annealing times and temperatures varied depending on the primer set (20-80 s, 45-48 °C) for the first primer pair and 30 s annealing for the second primer pair at 45 °C. Annealing was followed by a 1 min extension at 72 °C and a final extension at 72 °C varying between 5-10 min based on primer set. PCR products were all run on a 1% agarose gel stained with GelRed and purified using EXOFAP (EXO1 and FastAP) or via gel excision. Sequencing reactions were performed using 3.2 mM of respective primers and the BigDye TM terminator v. 3.1, and sequencing was done with an ABI Prism 3730XL automated sequencer. Forward and reverse reads were sequenced to achieve the greatest base calling accuracy for each species and targeted gene fragment. Sequence chromatograms in forward and reverse directions were trimmed (at an error probability limit of 0.05). Chromatograms were then assembled and edited by eye using Geneious 10 (51). Base calling while editing was made using the highest confidence score for any given base on one of the two chromatograms. All assembled chromatograms resulted in >90% high quality base pair reads with a mean Phred quality score ≥ 40. Assembled sequences were saved and exported as a fasta file. Each fasta file from targeted gene sequences was checked for contamination using the BLAST (52) function from GenBank. BLAST results that showed >85% sequence identity and a query cover of >60% to those belonging to Porifera were exported to Geneious 10 and aligned using the ClustalW function with default parameters. Samples showing ≥ 1% sequence differences along with unique morphological features were classified as different operational taxonomic units (OTUs). OTU abundance was measured as presence or absence of each OTU per side of plate, where values ranged from 0 to 6 for each OTU.

Sponge species richness was analyzed via ANOVA with temperature and pH as fixed factors, and header tank as a nested factor, followed by TukeyHSD *post hocs*.

Crustose coralline algae (CCA): In order to assess species richness, CCA were preliminarily sorted into parataxonomic units (53, 54) using a combination of surface morphology, and histological characters. Surface characters included texture (e.g., tessellation), color, conceptacle type, size, and shape. Histological information included presence of secondary pit connections and/or cell fusions, thallus thickness, conceptacle type, shape and structure, and size and morphology of epithelial cells. For the surface of each plate of the settlement tiles, samples of each species, as identified by eye, were lightly brushed across the surface with a soft toothbrush to remove visible epibionts and biofilm. Samples were removed with a chisel blade and quick dried in silica gel with indicator (55). Samples were further cleaned of epibionts under a

dissecting microscope. When sufficient sample was available, reproductive material was saved for histology.

Internal morphological characters were identified using histology (56). Specimens were prepared for histology by fixing in formalin for 24 hr, then decalcified in three changes of 0.6 M HNO_3 for a cumulative 5 hours soak time. Samples were dehydrated in a graded series of denatured alcohol (1 hr in each 10% increment, from 50% to 100%). Using JB-4® plastic embedding media (glycol methacrylate) and following kit instructions, specimens were soaked in two changes of infiltration solution (3 hr and 12 hr, respectively), transferred into embedding capsules (BEEM®, size 00), and embedded in catalyzed resin. From each embedded specimen, 10 sections (approximately 7 µm thick) were obtained by serial sectioning on an MT1 Porter-Blum microtome outfitted with a glass knife. The sections were floated on distilled water droplets distributed on microscope slides and then dried on a "warmer" (an electric griddle). Tissue sections (now affixed to the slides) were stained in a 0.5% solution of Toluidine Blue in water for 60 s. Excess stain was removed with a gentle stream of tap water, slides were rinsed in distilled water, and again dried on the "warmer". Coverslips were affixed to slides with Permount microscope slide mounting medium. CCA species richness was analyzed via ANOVA with temperature and pH as fixed factors, and header tank as a nested factor, followed by a TukeyHSD *post hoc*.

Water column microbes

Mesocosms were sampled on 26 July 2018. A peristaltic pump was used to flush 350 mL of sample over a 0.22 µm sterivex filter cartridge (47mm diameter equivalent, Sterivex, Millipore, Maryland) placed on ice and frozen at -80 °C within 4 hours of collection. Sterivex cartridges were cracked and filters transferred into Qiagen DNeasy PowerSoil Bead Tubes (Qiagen 12888-100) using a sterile scalpel and forceps. DNA was extracted following the DNeasy PowerSoil Kit protocol and eluted DNA was stored at -80 °C. Amplicon libraries targeting the 16S SSU rRNA V3-V4 hypervariable regions were generated from a single round of PCR using dual index 341F and 785R primers (57) and 1 µL genomic DNA template following standard reagents and PCR and library pooling conditions outlined by(58). A Zymobiomics mock community (Zymo D6306) was included as a positive control while PCR grade water was used as a negative control. Libraries were sequenced using an Illumina MiSeq V3 600 cycle run at the University of Hawaiʻi at Mānoa Advanced Studies in Genomics, Proteomics and Bioinformatics core facility. Raw sequencing reads were processed using mothur v1.41.3 (59). Following demultiplexing, contig assembly, and quality filtering, sequences were aligned to the silva rRNA database v132 (60). Sequences were denoised and then classified (61) within the mothur software environment. All sequences classified as Chloroplasts, Mitochondria, or as an Unknown domain were removed and each sample was subsampled to 6785 sequences. OTUs were clustered at 97% and rare OTUs were eliminated by removing any OTU that did not have a minimum relative abundance of 10^{-6} across all samples. Weighted unifrac community distance matrices were generated in mothur for multivariate comparisons of beta diversity from this pooled dataset. Microbial richness was examined via ANOVA with temperature and pH as fixed factors and header tank as a nested factor. Demultiplexed sequencing reads from the water column microbial samples analyzed in this study have been deposited in the NCBI Sequence Read Archive (SRA) under the BioProject Accession: PRJNA1154321

Fleshy algae

Small subsamples of the fleshy algae species present in each mesocosm were collected in July 2018. Each frondose and turf sample was placed in a plastic bag with sea water from the mesocosm and transported to the Botany Department at UH Mānoa in a cooler for further identification (62). Permanent slides of macroalgae and turf were made within a day of collection. All slides were identified using an Olympus BH-2 compound microscope and keys contained in prior work (63, 64) and updated via algaebase.org (65). Fleshy algae species richness was analyzed via ANOVA with temperature and pH as fixed factors, and header tank as a nested factor.

Overall species richness

Proportional changes in species richness for each of the datasets (sponges, CCA, and metabarcoding of metazoans from settlement tiles, coral-associated microbes, water column-associated microbes, coral-associated algal endosymbionts, fleshy algae, and corals) were calculated as the mean species richness for each group in each treatment relative to the maximum mean observed richness for that group in any of the four treatments. Analyses were performed by fitting a generalized linear model (GLM) with binomial error distribution, with temperature and pH as fixed effects. Significance of each factor was evaluated with LRTs. Analyses were performed using the R packages lme4 and lsmeans in R v.3.5.2 (81–83).

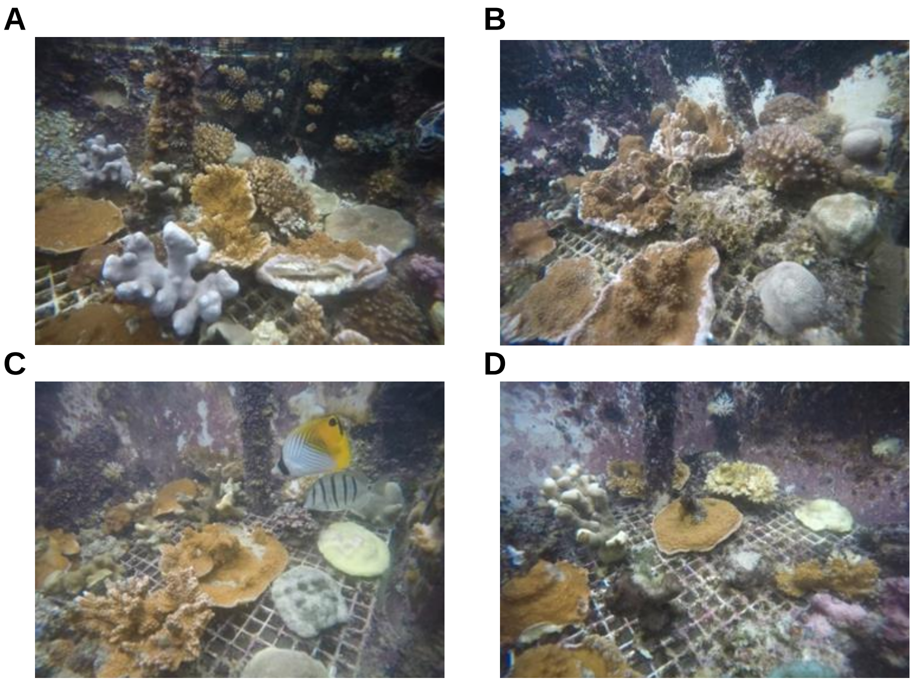

Fig. S1. Representative photos of the mesocosms after nearly 2 years of exposure under treatment conditions. Photos are from the (**A**) control, (**B**) ocean acidification, (**C**) ocean warming, and (**D**) combined future ocean treatments. Photos by authors.

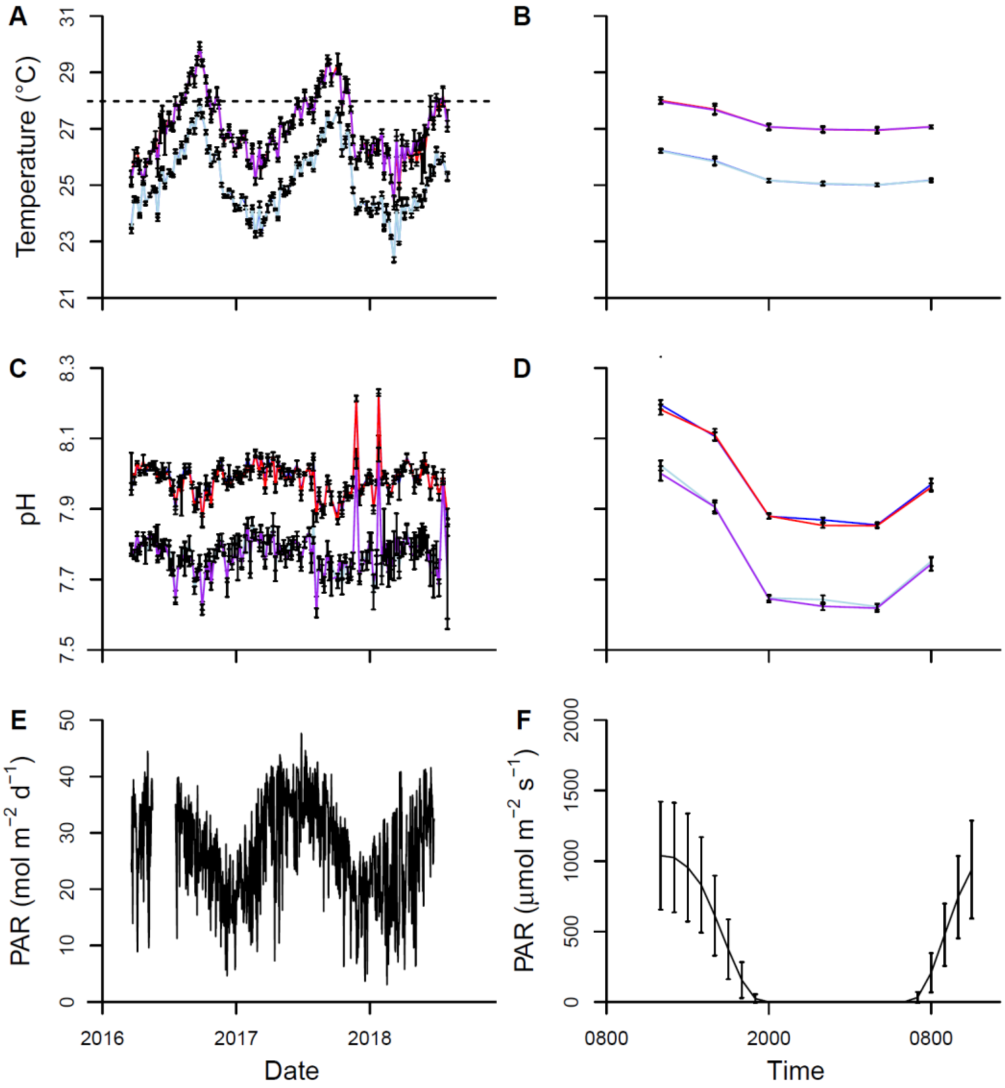

Fig. S2. Environmental data from the mesocosm experiment. Panels show time series of temperature (**A,B**) and pH (reported on the Total hydrogen ion scale) (**C,D**), as well as photosynthetically active radiation (PAR) (**E,F**) as daily means over the course of the experiment (**A,C)** and hourly means over the diel cycle (**B,D,F**) for the control (blue), ocean acidification (light blue), ocean warming (red), and combined future ocean (purple) treatments (individual lines not visible where they overlap). Horizontal dashed line (**A**) shows the nominal coral bleaching threshold for the Main Hawaiian islands. Temperature and pH are derived from weekly samples collected at 1200 hr local time as well as the mean of samples collected every 4 hr over the diel cycle once per month. Irradiance data are daily integral values (**E**), or the mean of hourly values (**F**). All data (except **E**) shown as mean ± SD. Error shown as vertical black bars (not visible where smaller than the line thickness). Mesocosms were covered in 30% shade cloth to replicate irradiance at mean collection depth (2 m). Maximum instantaneous irradiance was about 1730 µmol m^{-2} s^{-1} in the mesocosms and 2470 µmol m^{-2} s^{-1} in the air. Water circulation in the mesocosms was provided by seawater pumps (10-15 cm s^{-1}) and seawater turnover rate was 1 hr. The two spikes in pH during winter 2017/2018 are due to temporary (1 day) interruptions in the seawater flow through rate. See Table 1 for additional chemistry data, and see the Supplementary Information for additional details.

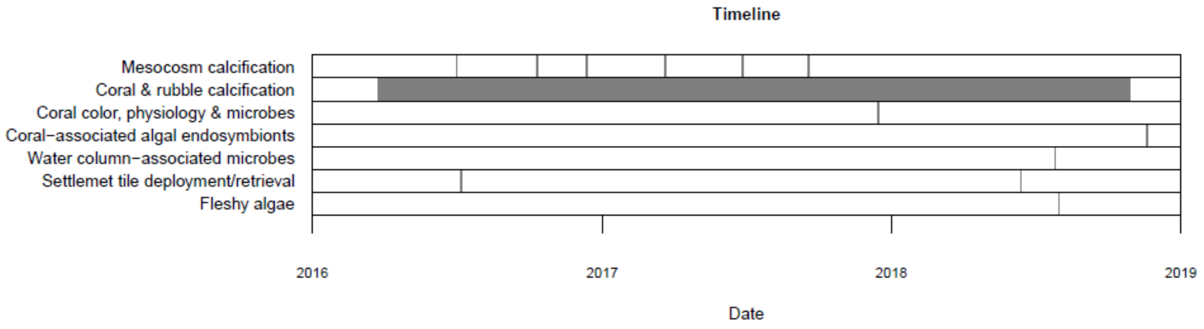

Fig. S3. Timeline of the experiment. Vertical lines in each row show sampling dates for each measurement and the gray bar indicates the integration period for the coral and rubble calcification measurements.

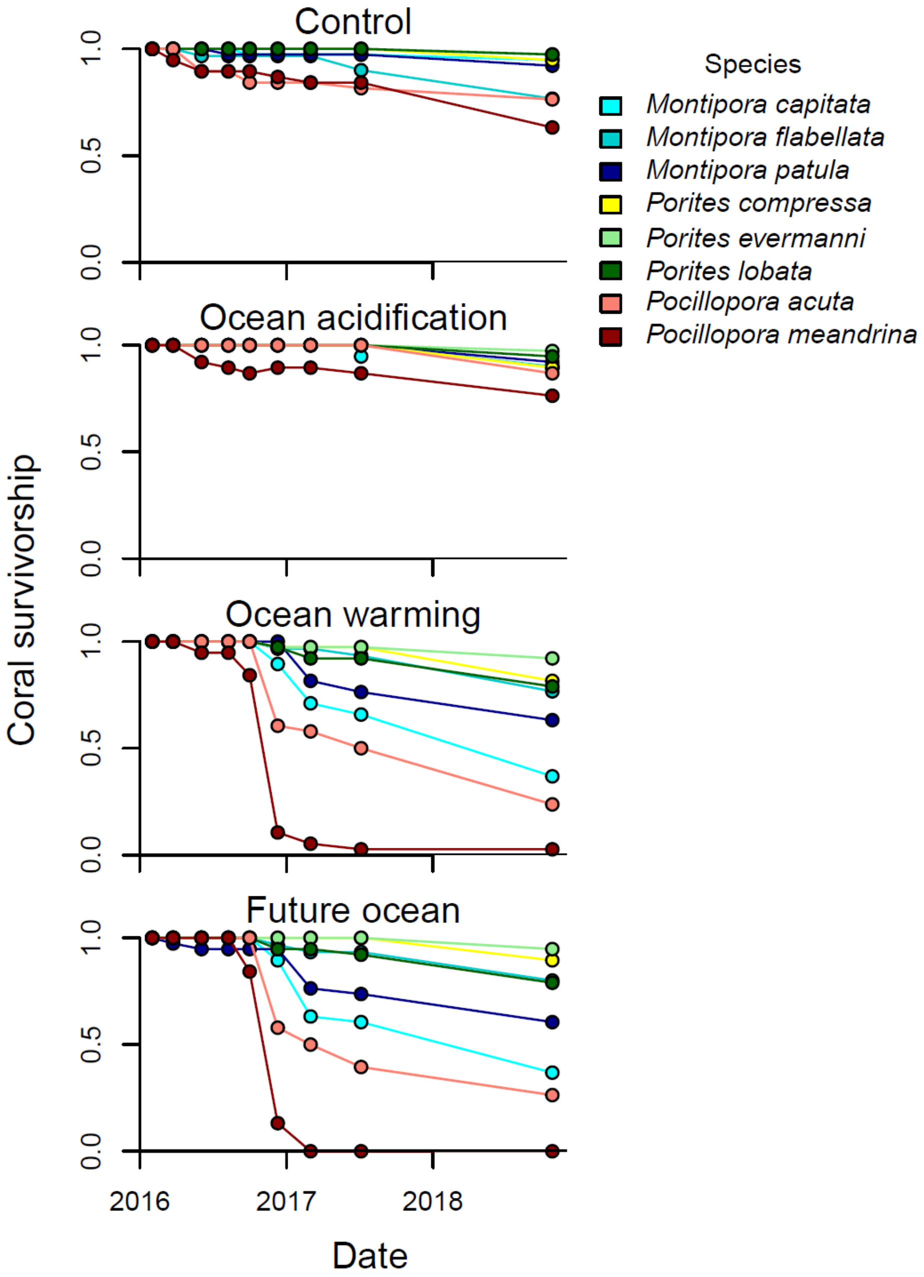

Fig. S4. Coral survivorship. Coral survivorship is shown according to species in each treatment, as indicated. Data shown as treatment means; error bars omitted for clarity.

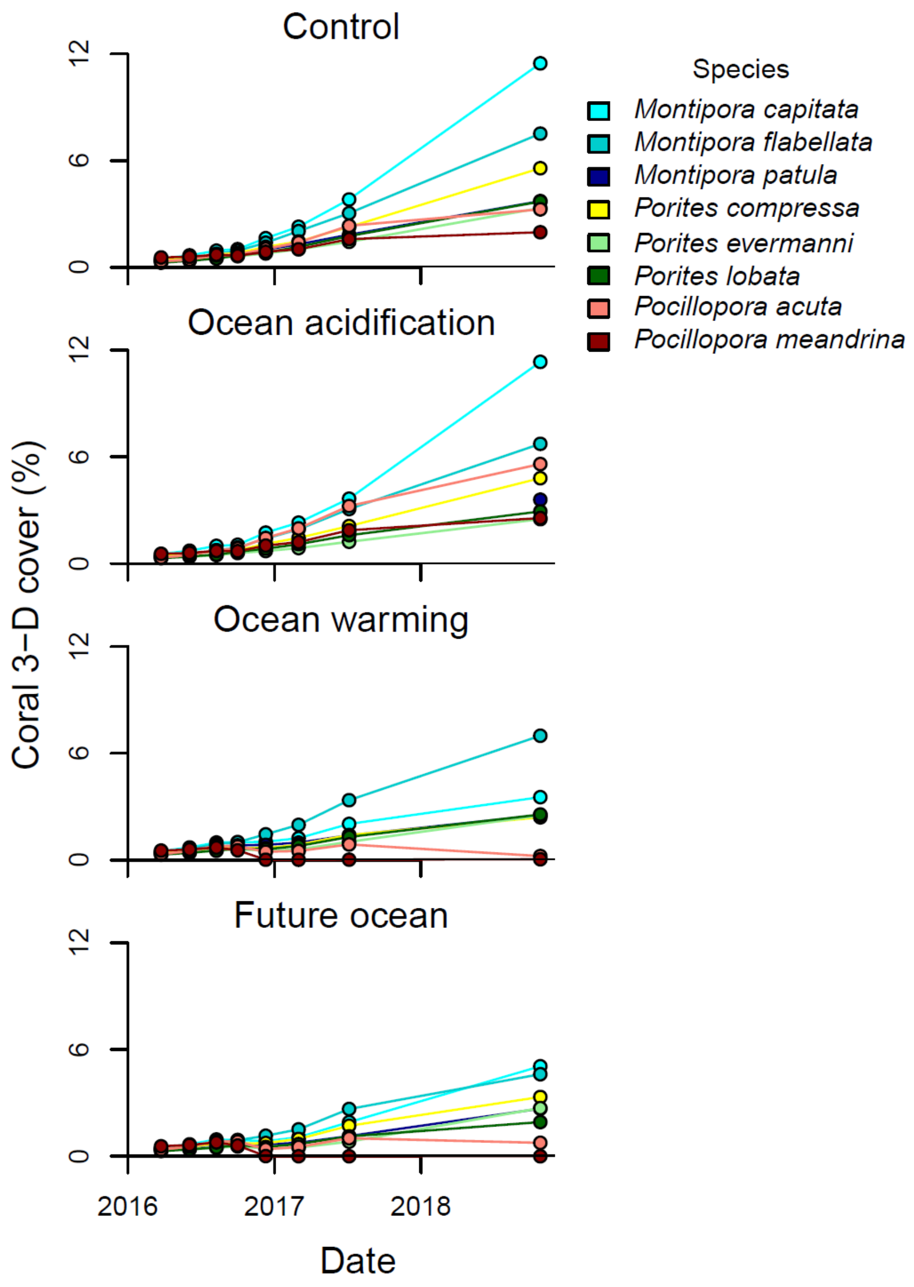

Fig. S5. Coral 3-D cover. Coral 3-D cover is shown according to species in each treatment and normalized to the initial 3-D surface area of the uncolonized mesocosms ($0.88m^2$), as indicated. Data shown as treatment means; error bars omitted for clarity.

Table S1. Likelihood ratio test results for treatment effects on coral survivorship and overall species richness. Results are based on GLMM model fits. For all factors, degrees of freedom = 1. Overall species richness is based on equally weighted results from corals, sponges, metazoans from settlement tiles, coral-associated microbes, coral-associated algal symbionts, water-column associated microbes, CCA, and fleshy algae, and exhibited no treatment effects.

**Response**	**P-value**
**Coral survivorship**	
Fixed effects	
Temp	**<0.001**
pH	0.332
Temp×pH	0.371
Random effects	
(1|Header)	0.938
(1|Mesocosm)	0.712

Table S2. ANOVA test results for treatment effects on coral 3-D cover, calcification, benthic cover on settlement tiles, and species richness. There were not significant treatment effects on the benthic cover of sediment, serpulid worms, or sponges, nor were there significant effects on the species richness of water-column associated microbes, coral-associated algal symbionts, CCA, or fleshy algae. P-values in bold are significant at alpha=0.05. Df=degrees of freedom, SS=sum of squares, MS=mean sum of squares.

**Response**	**Df**	**SS**	**MS**	**F**	**p**
**<u>Coral 3-D cover</u>**					
Temp	1	0.3763	0.3763	70.888	**<0.001**
pH	1	0.0000	0.0000	0.001	0.977
Temp×pH	1	0.0002	0.0002	0.029	0.866
Header(Temp×pH)	4	0.0237	0.0059	1.115	0.367
Residuals	32	0.1699	0.0053		
**<u>Calcification</u>**					
<u>Mesocosms</u>					
Temp	1	48.96	48.96	88.521	**<0.001**
pH	1	24.38	24.38	44.076	**<0.001**
Temp×pH	1	0.01	0.01	0.025	0.874
Header(Temp×pH)	4	7.14	1.78	3.226	**0.025**
Residuals	32	17.70	0.55		
<u>Corals</u>					
Temp	1	28.282	28.282	163.765	**<0.001**
pH	1	0.004	0.004	0.020	0.887
Temp×pH	1	0.039	0.039	0.227	0.637
Header(Temp×pH)	4	1.688	0.422	2.444	0.067
Residuals	32	5.526	0.173		
<u>Rubble</u>					
Temp	1	0.61	0.613	0.499	0.485
pH	1	27.84	27.841	22.667	**<0.001**
Temp×pH	1	0.05	0.054	0.044	0.836
Header(Temp×pH)	4	2.58	0.644	0.524	0.719
Residuals	32	39.30	1.228		
**<u>Benthic cover</u>**					
<u>Vermetid gastropods</u>					
Temp	1	0.01991	0.019907	7.130	**0.017**
pH	1	0.02673	0.026729	9.574	**<0.01**
Temp×pH	1	0.00809	0.008086	2.896	0.108
Header(Temp×pH)	4	0.01098	0.002744	0.983	0.445
Residuals	16	0.04467	0.002792		
<u>Biofilm/turf algae</u>					
Temp	1	0.01966	0.019659	4.917	**0.041**
pH	1	0.01320	0.013197	3.301	0.088
Temp×pH	1	0.00674	0.006740	1.686	0.213
Header(Temp×pH)	4	0.00953	0.002382	0.596	0.671
Residuals	16	0.06398	0.003998		
<u>CCA</u>					
Temp	1	0.03060	0.030598	4.679	**0.046**
pH	1	0.01348	0.013480	2.061	0.170
Temp×pH	1	0.00006	0.000058	0.009	0.926

Header(Temp×pH)	4	0.01288	0.003219	0.492	0.742
Residuals	16	0.10463	0.006539		
<u>Motile fauna</u>					
Temp	1	0.02321	0.023206	8.431	**0.010**
pH	1	0.00833	0.008327	3.026	0.101
Temp×pH	1	0.00016	0.000163	0.059	0.812
Header(Temp×pH)	4	0.00966	0.002414	0.877	0.499
Residuals	16	0.04404	0.002752		
**<u>Species richness</u>**					
<u>Sponges</u>					
Temp	1	3.37	3.37	0.463	0.506
pH	1	35.04	35.04	4.806	**0.044**
Temp×pH	1	2.04	2.04	0.280	0.604
Header(Temp×pH)	4	44.83	11.21	1.537	0.239
Residuals	16	116.67	7.29		
<u>Corals</u>					
Temp	1	21.025	21.025	46.722	**<0.001**
pH	1	0.025	0.025	0.056	0.815
Temp×pH	1	0.225	0.225	0.500	0.485
Header(Temp×pH)	4	1.300	0.325	0.722	0.583
Residuals	32	14.400	0.450		
<u>Coral-associated microbes</u>					
Temp	1	304,960	304,960	4.148	**0.043**
pH	1	137,310	137,310	1.868	0.173
Temp×pH	1	50,826	50,826	0.691	0.407
Header(Temp×pH)	4	546,551	136,638	1.859	0.119
Mesocosm(Header(Temp×pH)	32	1,283,217	40,101	0.546	0.978

Table S3. PERMANOVA test results for treatment effects on benthic community structure on settlement tiles, and coral-associated microbial communities. Water column-associated microbial communities showed no treatment effects.

Response	Df	SS	MS	F	P
**Benthic community structure**					
Temp	1	0.032352	0.032352	3.4011	**0.016**
pH	1	0.019522	0.019522	2.50523	0.091
Temp×pH	1	0.009421	0.009421	0.9905	0.435
Header(Temp×pH)	4	0.066785	0.016696	1.7553	.067
Residuals	16	0.15191	0.009512		
Total	23	0.280271			

Table S4. Results of Kruskal-Wallis tests for treatment effects on benthic cover on settlement tiles (ARMS). Benthic cover of anemones, bivalves, bivalve shells, solitary tunicates, uncolonized space, unavailable space, and fleshy algae showed no treatment effects. P-values show in bold are significant at alpha=0.05.

Response	Chi-square	Df	p
**Benthic cover**			
Encrusting green algae	9.8182	3	**0.020**

Table S5. Power analyses for those groups on settlement tiles (ARMS) which did not exhibit significant treatment effects. The minimum detectable effect size at a power of 0.95 was 1.69, or a difference in benthic over among treatments of about 0.5%. N is sample size and Df is degrees of freedom.

**Group**	**Effect size**	**N**	**Df**	**Power**
Anemone	0.583	6	3	0.195
Bivalve	0.408	6	3	0.116
Bivalve (empty)	0.818	6	3	0.360
Solitary tunicate	0.619	6	3	0.217
Uncolonized space	0.984	6	3	0.504
Unavailable space	0.424	6	3	0.121
Serpulid worm	0.709	6	3	0.276
Sponge	0.352	6	3	0.098
Sediment	0.442	6	3	0.128
Fleshy algae	0.753	6	3	0.309

SI References

1. K. E. F. Shamberger, *et al.*, Calcification and organic production on a Hawaiian coral reef. *Mar. Chem.* **127**, 64–75 (2011).
2. K. Bathen, "A descriptive study of the physical oceanography of Kane'ohe Bay." in (1968).
3. C. P. Jury, F. I. M. Thomas, M. J. Atkinson, R. J. Toonen, Buffer Capacity, Ecosystem Feedbacks, and Seawater Chemistry under Global Change. *Water* **5**, 1303–1325 (2013).
4. C. P. Jury, M. N. Delano, R. J. Toonen, High heritability of coral calcification rates and evolutionary potential under ocean acidification. *Sci. Rep.* **9**, 20419 (2019).
5. Ò. Guadayol, N. J. Silbiger, M. J. Donahue, F. I. M. Thomas, Patterns in Temporal Variability of Temperature, Oxygen and pH along an Environmental Gradient in a Coral Reef. *PLOS ONE* **9**, e85213 (2014).
6. A. G. Dickson, C. L. Sabine, J. R. Christian, "Guide to Best Practices for Ocean CO2 Measurements." (North Pacific Marine Science Organization, 2007).
7. OCADS - Program Developed for CO_2 System Calculations. Available at: https://www.ncei.noaa.gov/access/ocean-carbon-data-system/oceans/CO2SYS/co2rprt.html [Accessed 22 March 2021].
8. P. L. Jokiel, *et al.*, Ocean acidification and calcifying reef organisms: a mesocosm investigation. *Coral Reefs* **27**, 473–483 (2008).
9. S. Comeau, *et al.*, Flow-driven micro-scale pH variability affects the physiology of corals and coralline algae under ocean acidification. *Sci. Rep.* **9**, 12829 (2019).
10. S. G. Dove, K. T. Brown, A. Van Den Heuvel, A. Chai, O. Hoegh-Guldberg, Ocean warming and acidification uncouple calcification from calcifier biomass which accelerates coral reef decline. *Commun. Earth Environ.* **1**, 1–9 (2020).
11. D. Riddle, Product Review: Maxi-Jet Pro Series Pumps. *Reefs.com*. Available at: https://reefs.com/magazine/product-review-maxi-jet-pro-series-pumps/ [Accessed 22 March 2021].
12. K. D. Gorospe, *et al.*, Local Biomass Baselines and the Recovery Potential for Hawaiian Coral Reef Fish Communities. *Front. Mar. Sci.* **5** (2018).
13. W. M. Hamner, M. S. Jones, J. H. Carleton, I. R. Hauri, D. McB. Williams, Zooplankton, Planktivorous Fish, and Water Currents on a Windward Reef Face: Great Barrier Reef, Australia. *Bull. Mar. Sci.* **42**, 459–479 (1988).
14. T. D. Clark, *et al.*, Ocean acidification does not impair the behaviour of coral reef fishes. *Nature* **577**, 370–375 (2020).
15. E. C. Franklin, P. L. Jokiel, M. J. Donahue, Predictive modeling of coral distribution and abundance in the Hawaiian Islands. *Mar. Ecol. Prog. Ser.* **481**, 121–132 (2013).
16. K. S. Rodgers, P. L. Jokiel, E. K. Brown, S. Hau, R. Sparks, Over a Decade of Change in Spatial and Temporal Dynamics of Hawaiian Coral Reef Communities1. *Pac. Sci.* **69**, 1–13 (2015).
17. Z. H. Forsman, D. J. Barshis, C. L. Hunter, R. J. Toonen, Shape-shifting corals: Molecular markers show morphology is evolutionarily plastic in Porites. *BMC Evol. Biol.* **9**, 45 (2009).
18. J. N. Boulay, M. E. Hellberg, J. Cortés, I. B. Baums, Unrecognized coral species diversity masks differences in functional ecology. *Proc. R. Soc. B Biol. Sci.* **281**, 20131580 (2014).
19. J. E. N. Veron, M. G. Stafford-Smith, E. Turak, L. M. DeVantier, *Corals of the World,* (2016).
20. J. Stolarski, *et al.*, The ancient evolutionary origins of Scleractinia revealed by azooxanthellate corals. *BMC Evol. Biol.* **11**, 316 (2011).
21. E. S. Darling, L. Alvarez-Filip, T. A. Oliver, T. R. McClanahan, I. M. Côté, Evaluating life-history strategies of reef corals from species traits. *Ecol. Lett.* **15**, 1378–1386 (2012).
22. NOAA Coral Reef Watch, NOAA Coral Reef Watch Version 3.1 Daily Global 5-km Satellite Coral Bleaching Degree Heating Week Product. [Preprint] (2018). [Accessed 1 February 2020].
23. A. Cros, R. J. Toonen, S. W. Davies, S. A. Karl, Population genetic structure between Yap and Palau for the coral Acropora hyacinthus. *PeerJ* **4**, e2330 (2016).

24. A. Cros, R. J. Toonen, M. J. Donahue, S. A. Karl, Connecting Palau's marine protected areas: a population genetic approach to conservation. *Coral Reefs* **36**, 735–748 (2017).
25. G. T. Concepcion, N. R. Polato, I. B. Baums, R. J. Toonen, Development of microsatellite markers from four Hawaiian corals: Acropora cytherea, Fungia scutaria, Montipora capitata and Porites lobata. *Conserv. Genet. Resour.* **2**, 11–15 (2010).
26. B. C. Faircloth, T. C. Glenn, Not All Sequence Tags Are Created Equal: Designing and Validating Sequence Identification Tags Robust to Indels. *PLOS ONE* **7**, e42543 (2012).
27. P. Meirmans, GenoDive version 2.b14.
28. K. A. Selkoe, R. J. Toonen, Microsatellites for ecologists: a practical guide to using and evaluating microsatellite markers. *Ecol. Lett.* **9**, 615–629 (2006).
29. P. G. Meirmans, P. H. V. Tienderen, genotype and genodive: two programs for the analysis of genetic diversity of asexual organisms. *Mol. Ecol. Notes* **4**, 792–794 (2004).
30. C. A. Schneider, W. S. Rasband, K. W. Eliceiri, NIH Image to ImageJ: 25 years of image analysis. *Nat. Methods* **9**, 671–675 (2012).
31. D. Bates, M. Mächler, B. Bolker, S. Walker, Fitting Linear Mixed-Effects Models Using lme4. *J. Stat. Softw.* **67**, 1–48 (2015).
32. R. V. Lenth, Least-Squares Means: The R Package lsmeans. *J. Stat. Softw.* **69**, 1–33 (2016).
33. R Core Team, R: A language and environment for statistical computing. [Preprint] (2018). Available at: https://www.R-project.org/.
34. P. L. Jokiel, J. E. Maragos, L. Franzisket, D. R. Stoddart, R. E. Johannes, Coral reefs: research methods. Coral growth: buoyant weight technique. *UNESCO Paris* 529–541 (1978).
35. A. J. Andersson, *et al.*, Net Loss of CaCO3 from a subtropical calcifying community due to seawater acidification: Mesocosm-scale experimental evidence. *Biogeosciences* **6**, 13 (2009).
36. M. R. Gaither, Z. Szabó, M. W. Crepeau, C. E. Bird, R. J. Toonen, Preservation of corals in salt-saturated DMSO buffer is superior to ethanol for PCR experiments. *Coral Reefs* **30**, 329–333 (2011).
37. M. R. de Souza, *et al.*, Community composition of coral-associated Symbiodiniaceae differs across fine-scale environmental gradients in Kāneʻohe Bay. *R. Soc. Open Sci.* **9**, 212042 (2022).
38. M. Martin, Cutadapt removes adapter sequences from high-throughput sequencing reads. *EMBnet.journal* **17**, 3 (2011).
39. B. C. C. Hume, *et al.*, SymPortal: A novel analytical framework and platform for coral algal symbiont next-generation sequencing *ITS2* profiling. *Mol. Ecol. Resour.* **19**, 1063–1080 (2019).
40. J. Okansen, *et al.*, vegan: Community ecology package. R package version 2.5. 4. 2019. [Preprint] (2019).
41. M. D. Cáceres, P. Legendre, Associations between species and groups of sites: indices and statistical inference. *Ecology* **90**, 3566–3574 (2009).
42. pwr: Basic functions for power analysis (2017).
43. M. A. Timmers, J. Vicente, M. Webb, C. P. Jury, R. J. Toonen, Sponging up diversity: Evaluating metabarcoding performance for a taxonomically challenging phylum within a complex cryptobenthic community. *Environ. DNA* **n/a**.
44. J. Vicente, *et al.*, Unveiling hidden sponge biodiversity within the Hawaiian reef cryptofauna. *Coral Reefs* (2021). https://doi.org/10.1007/s00338-021-02109-7.
45. O. Folmer, M. Black, W. Hoeh, R. Lutz, R. Vrijenhoek, DNA primers for amplification of mitochondrial cytochrome c oxidase subunit I from diverse metazoan invertebrates. *Mol. Mar. Biol. Biotechnol.* **3**, 294–299 (1994).
46. L.-Y. Chuang, Y.-H. Cheng, C.-H. Yang, Specific primer design for the polymerase chain reaction. *Biotechnol. Lett.* **35**, 1541–1549 (2013).
47. J. Geller, C. Meyer, M. Parker, H. Hawk, Redesign of PCR primers for mitochondrial cytochrome c oxidase subunit I for marine invertebrates and application in all-taxa biotic surveys. *Mol. Ecol. Resour.* **13**, 851–861 (2013).

48. M. Leray, *et al.*, A new versatile primer set targeting a short fragment of the mitochondrial COI region for metabarcoding metazoan diversity: application for characterizing coral reef fish gut contents. *Front. Zool.* **10**, 34 (2013).
49. M. Medina, A. G. Collins, J. D. Silberman, M. L. Sogin, Evaluating hypotheses of basal animal phylogeny using complete sequences of large and small subunit rRNA. *Proc. Natl. Acad. Sci.* **98**, 9707–9712 (2001).
50. C. Chombard, N. Boury-Esnault, S. Tillier, Reassessment of Homology of Morphological Characters in Tetractinellid Sponges Based on Molecular Data. *Syst. Biol.* **47**, 351–366 (1998).
51. M. Kearse, *et al.*, Geneious Basic: An integrated and extendable desktop software platform for the organization and analysis of sequence data. *Bioinformatics* **28**, 1647–1649 (2012).
52. S. F. Altschul, W. Gish, W. Miller, E. W. Myers, D. J. Lipman, Basic local alignment search tool. *J. Mol. Biol.* **215**, 403–410 (1990).
53. Y. Basset, V. Novotny, S. E. Miller, R. Pyle, Quantifying Biodiversity: Experience with Parataxonomists and Digital Photography in Papua New Guinea and Guyana. *BioScience* **50**, 899–908 (2000).
54. I. Oliver, A. J. Beattie, A Possible Method for the Rapid Assessment of Biodiversity. *Conserv. Biol.* **7**, 562–568 (1993).
55. B. A. Twist, *et al.*, High diversity of coralline algae in New Zealand revealed: Knowledge gaps and implications for future research. *PLOS ONE* **14**, e0225645 (2019).
56. S. Kaleb, G. Alongi, A. Falace, “Coralline algae preparatuion for scanning electron microscope and optical microscopy” in *Protocols for Macroalgae Research*, (CRC Press, 2018), pp. 411–428.
57. A. Klindworth, *et al.*, Evaluation of general 16S ribosomal RNA gene PCR primers for classical and next-generation sequencing-based diversity studies. *Nucleic Acids Res.* **41**, e1–e1 (2013).
58. J. J. Kozich, S. L. Westcott, N. T. Baxter, S. K. Highlander, P. D. Schloss, Development of a Dual-Index Sequencing Strategy and Curation Pipeline for Analyzing Amplicon Sequence Data on the MiSeq Illumina Sequencing Platform. *Appl. Environ. Microbiol.* **79**, 5112–5120 (2013).
59. P. D. Schloss, *et al.*, Introducing mothur: Open-Source, Platform-Independent, Community-Supported Software for Describing and Comparing Microbial Communities. *Appl. Environ. Microbiol.* **75**, 7537–7541 (2009).
60. C. Quast, *et al.*, The SILVA ribosomal RNA gene database project: improved data processing and web-based tools. *Nucleic Acids Res.* **41**, D590–D596 (2013).
61. J. T. L. Wang, *et al.*, New Techniques for DNA Sequence Classification. *J. Comput. Biol.* **6**, 209–218 (1999).
62. R. T. Tsuda, I. A. Abbott, “Collection, handling, preservation, and logistics” in *Ecological Field Methods: Macroalgae. Handbook of Phycological Methods*, (Cambridge University Press, 1985), pp. 67–68.
63. I. A. Abbott, J. M. Huisman, *Marine green and brown algae of the Hawaiian Islands* (Bishiop Museum Press, 2004).
64. I. A. Abbott, *Marine Red Algae of the Hawaiian Islands* (Bishop Museum Press, 1999).
65. M. D. Guiry, G. M. Guiry, AlgaeBase. *AlgaeBase* (2008).
